# Investigating Macrophages Plasticity Following Tumour–Immune Interactions During Oncolytic Therapies

**DOI:** 10.1007/s10441-019-09357-9

**Published:** 2019-08-13

**Authors:** R. Eftimie, G. Eftimie

**Affiliations:** 1 Division of Mathematics, University of Dundee, Dundee, DD1 4HN UK; 2 Hopital Privé de La Miotte, 90002 Belfort, France

**Keywords:** Mathematical model, Oncolytic viruses, M1 macrophages, M2 macrophages, $\hbox {CD8}^{+}$ T cells

## Abstract

Over the last few years, oncolytic virus therapy has been recognised as a promising approach in cancer treatment, due to the potential of these viruses to induce systemic anti-tumour immunity and selectively killing tumour cells. However, the effectiveness of these viruses depends significantly on their interactions with the host immune responses, both innate (e.g., macrophages, which accumulate in high numbers inside solid tumours) and adaptive (e.g., $\hbox {CD8}^{+}$ T cells). In this article, we consider a mathematical approach to investigate the possible outcomes of the complex interactions between two extreme types of macrophages (M1 and M2 cells), effector $\hbox {CD8}^{+}$ T cells and an oncolytic Vesicular Stomatitis Virus (VSV), on the growth/elimination of B16F10 melanoma. We discuss, in terms of VSV, $\hbox {CD8}^{+}$ and macrophages levels, two different types of immune responses which could ensure tumour control and eventual elimination. We show that both innate and adaptive anti-tumour immune responses, as well as the oncolytic virus, could be very important in delaying tumour relapse and eventually eliminating the tumour. Overall this study supports the use mathematical modelling to increase our understanding of the complex immune interaction following oncolytic virotherapies. However, the complexity of the model combined with a lack of sufficient data for model parametrisation has an impact on the possibility of making quantitative predictions.

## Introduction

The increase in the incidence of skin cancers, combined with the advances in understanding the molecular biological mechanisms involved in tumour progression and interactions between melanoma cells and immune cells, has led to the development of several immune strategies for the treatment of these cancers (Dharmadhikari et al. 2015). Among these strategies, the use of oncolytic virotherapies is emerging as an important approach in cancer treatment, due to their potential of inducing systemic anti-tumour immunity in addition to selectively killing cancer cells (Kaufman et al. 2016; Fukuhara et al. 2016; Filley and Dey 2017). In spite of current expectations that oncolytic virus therapy will become in the future a standard therapy option for all cancer patients (Fukuhara et al. 2016), there are still limitations of this therapy. The reduced effectiveness of the oncolytic viruses injected into cancer patients depends not only on the pathogenic nature of virally encoded genes, but also on the interactions between the virus and the host innate and adaptive immune responses (Melcher et al. 2011; Kaufman et al. 2016; Fukuhara et al. 2016; Filley and Dey 2017).

**Fig. 1:**
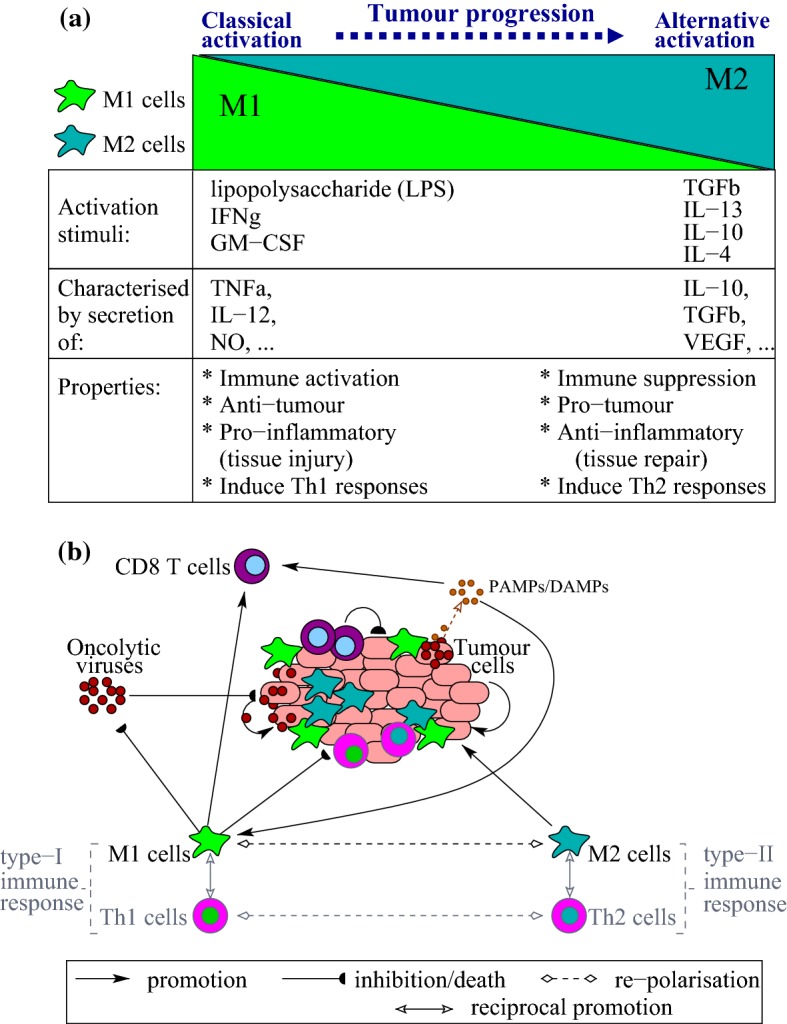
**a** Classification of macrophages phenotypes, where cells are considered as part of a continuum, with the two extreme phenotypes of macrophages polarisation being represented by the classically activated M1 cells and the alternatively activated M2 cells. Tumour progression induces a $\hbox {M1}\rightarrow \hbox {M2}$ polarisation (Mantovani and Sica 2010). Recent studies have also suggested a $\hbox {Th1}\rightarrow \hbox {Th2}$ polarisation during tumour progression [see, for example, Fig. 5 in Tatsumi et al. (2004)]. **b** Caricature description of the interactions between tumour cells, innate immunity (described here by M1 and M2 cells), adaptive immunity (described here mainly by the $\hbox {CD8}^{+}$ T cells), and oncolytic viruses. Oncolytic viruses can lead to the production of viral pathogen-associated molecular patterns (PAMPs) that can be presented on the surface of tumour cells or released in the microenvironment (Pol et al. 2012), or danger-associated molecular patterns (DAMPs) that are released in the microenviromnent following virus-induced tumour lysis and which can promote macrophages activation (Martin 2016). In this figure we show also the Th1 and Th2 $\hbox {CD4}^{+}$ T cells, which interact with the M1 and M2 cells generating type-I and type-II immune responses (Allavena and Mantovani 2012; Mills 2012)

Macrophages are one of the key innate immune cells involved in the regulation of anti-cancer immunotherapies, having either immuno-stimulatory or immuno-suppresive effects (Allavena and Mantovani 2012). These cells can display different phenotypes, in response to the type, concentration and longevity of exposure to stimulating agents (Cassetta et al. 2011). The two extreme macrophages phenotypes are represented by the M1 and M2 cells; see (Mantovani et al. 2002; Sica et al. 2008; Allavena and Mantovani 2012) and also Fig. 1. Note that this M1-M2 classification follows the Th1–Th2 ($\hbox {CD4}^{+}$ T cells) classification, since the M1 cells are stimulated by Th1 cytokines, and M2 cells are stimulated by Th2 cytokines (Allavena and Mantovani 2012). While it is accepted that the classically-activated M1 cells have anti-tumour properties and the alternatively-activated M2 cells have pro-tumour properties, many studies have shown that macrophages inside the tumour microenvironment have markers characterising mixed phenotypes (Mantovani and Sica 2010; Allavena and Mantovani 2012; Italiani and Boraschi 2014). Because of the heterogeneity and plasticity of macrophages, it is very difficult to predict their impact on oncolytic virotherapies (Jakeman et al. 2015; Denton et al. 2016). For example, it has been shown experimentally that the M2 macrophages can support these therapies through the suppression of the anti-viral immune response (Denton et al. 2016). The M1 macrophages may impede oncolytic therapies through the promotion of an anti-viral immune response that leads to viral clearance, but they also enhance the virus-mediated activation of the anti-tumour immune responses, which includes cells of the adaptive response such as $\hbox {CD8}^{+}$ T cells (Denton et al. 2016).

In addition to the anti-viral innate immunity triggered by macrophages, as well as by other innate immune cells (e.g., neutrophils, natural killer (NK) cells; see Filley and Dey 2017), oncolytic therapies can be impeded also by various adaptive immune responses (e.g., $\hbox {CD8}^{+}$ T cells; see Filley and Dey 2017; Melcher et al. 2011) and tumour/virus-induced cytokine production (e.g., IFN-$\gamma$, TNF-$\alpha$; see Filley and Dey 2017), which can have direct anti-viral activities as well as immunoregulatory activities (Filley and Dey 2017). These complex immune–virus interactions lead also to the development of the two main approaches on oncolytic therapies: the virocentric point of view (which sees the immune system as an obstacle to viral replication), and the immunocentric point of view (which focuses on the immunogenicity of the viruses and their roles in inducing effector immune responses that can eliminate disseminated tumour cells) (Alemany and Cascallo 2009; Russell and Peng 2017). To overcome the current limitations of virotherapies, which in most of the cases are applied to immunocompromised patients (Alemany and Cascallo 2009; Russell and Peng 2017), it is extremely important to investigate the complex interactions between oncolytic viruses and innate immunity (represented by pro-tumour/anti-tumour macrophages) as well as adaptive immunity (represented by $\hbox {CD8}^{+}$ T cells).

In this study, we focus on a highly metastatic cell line, B16F10 melanoma, which is also resistant against the $\hbox {CD8}^{+}$ cytotoxic T lymphocytes. Previous experimental studies with C57BL/6 mice have investigated the anti-tumour effects of $\hbox {CD8}^{+}$ T cells following the inoculation of Vesicular Stomatitis Virus (VSV) particles, which take advantage of the impaired interferon pathways in tumour cells (Hastie and Grdzelishvili 2012). In addition, experimental studies have shown that melanoma tumours can contain up to 30% macrophages (Hussein 2006), and hence the elucidation of the interaction mechanisms between macrophages and $\hbox {CD8}^{+}$ T cells is very important in the context of oncolytic therapies. Moreover, recent experimental results have emphasised the importance of macrophages on the motility and dissemination of murine melanoma cells (Roh-Johnson et al. 2017). However, to the best of our knowledge, not many experimental studies have investigated in a quantitative manner (i.e., showing time-series experimental data) the complex pro-tumour/anti-tumour effects of various innate and adaptive immune cells in the tumour microenvironment, following the administration of an oncolytic virus (e.g., VSV).

To address this, in our paper we aim to investigate at a theoretical level (i.e., using mathematical and computational approaches, combined with different published experimental data), the interactions between the M1/M2 macrophages, the $\hbox {CD8}^{+}$ T cells primed by the macrophages (although they can also be primed by the dendritic cells Pozzi et al. 2005), and the VSV particles injected in a B16F10 tumour. To keep the model relatively simple, we will ignore other innate immune cells in the tumour microenvironment, such as dendritic cells (DCs) (Pozzi et al. 2005), natural killer (NK) cells (Grundy et al. 2006), which have also been associated with increased tumour elimination (Grundy et al. 2006). We will also ignore the explicit dynamics of $\hbox {CD4}^{+}$ T helper cells, which have been shown to interact with the M1/M2 macrophages (Allavena and Mantovani 2012) and with the $\hbox {CD8}^{+}$ T cells (de Boer et al. 2003) during anti-tumour and anti-viral immune responses; see also Fig. 1b. However, we need to emphasise that due to the mirroring of the M1 and Th1 immune responses, as well as the M2 and Th2 responses, even if we talk about M1 and M2 macrophages we think of [as suggested in Mills (2012)] the more broad type-I immune responses (i.e., responses generated by M1 and Th1 cells) and type-II immune responses (i.e., responses generated by M2 and Th2 cells).

Mathematical and computational approaches have been widely used in the past to investigate the interactions between oncolytic viruses, tumour cells and immune cells (mainly cytotoxic T cells), with the overall aim of explaining existing observations or generating new testable hypotheses; see (Nowak and May 2000; Wodarz 2001; Wodarz and Komarova 2009; Friedman et al. 2006; Bajzer et al. 2008; Dingli et al. 2009; Paiva et al. 2009; Komarova and Wodarz 2010; Wu et al. 2004; Eftimie et al. 2011; Hofacre et al. 2012; Rommelfanger et al. 2012; Crivelli et al. 2012; Macnamara and Eftimie 2015; Kim et al. 2015; Malinzi et al. 2015; Eftimie et al. 2016; Eftimie and Hamam 2017) and the references therein. The majority of these models focus on the temporal evolution of viral titers and immune cell responses, thus being described by ordinary differential equations. Fewer models focus also on the spatial distribution of viruses inside solid tumours (Paiva et al. 2009; Hofacre et al. 2012; Malinzi et al. 2015; Timalsina et al. 2017; Malinzi et al. 2017). Other mathematical models have been derived to investigate the role of M1 and M2 macrophages (and other innate immune cells) on tumour dynamics, including B10F16 melanoma (Louzoun et al. 2014; den Breems and Eftimie 2016; Eftimie and Hamam 2017; Phan and Tian 2017). A few other models investigated the anti-tumour effects of the interactions between innate and adaptive immune responses (Louzoun et al. 2014; Eftimie et al. 2010), and even fewer models focused on the effects of innate and adaptive immunity on oncolytic viral therapies (Timalsina et al. 2017).

**Fig. 2:**
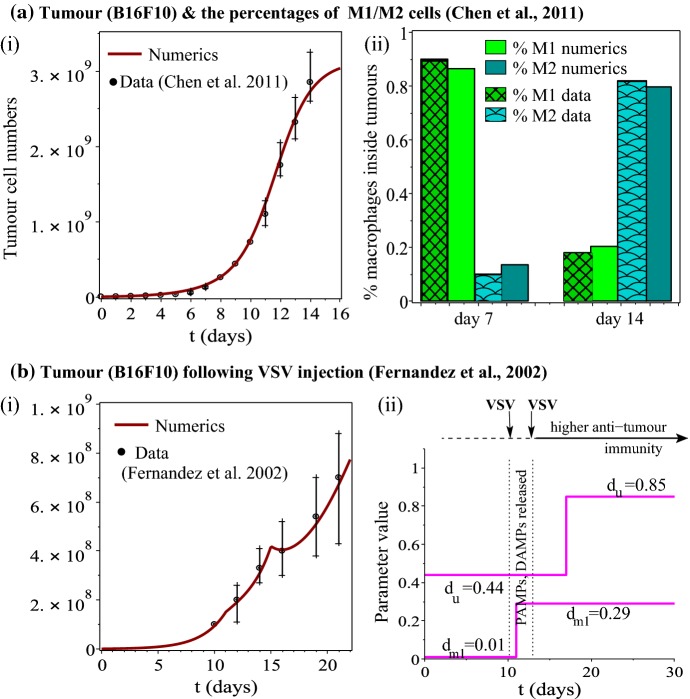
**a** (i) Growth of B16F10 tumour cells and (ii) the re-polarisation of M1 and M2 macrophages, as described by data re-drawn from Chen et al. (2011). Here the authors injected mice with $5\times 10^{6}$ tumour cells. For the numerical simulations we assume the following initial conditions: $x_{\text {u}}(0)=5\times 10^{6}$, $x_{\text {m1}}(0)=x_{\text {m2}}(0)=0$, $x_{\text {e}}(0)=0$; **b** Growth of B16F10 tumour cells in the presence of treatment with wild-type VSV, as described by data re-drawn from Fernandez et al. (2002). Here, the authors injected mice with $5\times 10^5$ tumour cells, and after palpable tumours were formed (of volumes $\approx 100$mm$^{3}$) the mice were treated twice (on days 10 and 13) with $2\times 10^{7}$ PFU of wild-type VSV. Mathematically, this VSV administration is described with the help of function $H(t)=2\times 10^{7}\left (Heaviside(t-10)\cdot Heaviside(11-t)+Heaviside(t-13)\cdot Heaviside(14-t) \right )$. Since virus injection leads to tumour cell killing and release from the lysed cells of tumour-associated antigens (TAAs), pathogen-associated molecular patterns (PAMPs) and danger-associated molecular patterns (DAMPs), which can activate macrophages within hours (Martin 2016; Melzer et al. 2017), and further lead to the activation of $\hbox {CD8}^{+}$ T cells within 4–7 days (following the release of tumour-associated antigens Diaz et al. 2007b; Melzer et al. 2017), here we consider two values for the anti-tumour killing rates $d_{u}$ and $d_{m1}$, for before and after the detection of TAAs/PAMPs/DAMPs: $d_{m1}=0.01$ for $t<11$ and $d_{m1}=0.29$ for $t>11$, and $d_{u}=0.44$ for $t<15$ (i.e., 4–5 days after VSV leads to the release of DAMPs) and $d_{u}=0.85$ for $t>15$; see sub-panel (b)(ii)

In the following, we use a mathematical approach to investigate the complex dynamics between the $\hbox {CD8}^{+}$ T cells and M1 and M2 macrophages in the tumour microenvironment, with the overall aim of proposing new hypotheses regarding the mechanisms that could improve the anti-tumour effect of oncolytic viruses. In Sect. 2, we start with the experimental protocol introduced in Fernandez et al. (2002), where a Vesicular Stomatitis Virus (VSV) is injected into the system on two different days (days 10 and 13 after the inoculation of B16F10 melanoma cells), and use it to introduce a new mathematical model that quantifies the levels of virus infected/uninfected tumour cells, the levels of M1 and M2 macrophages, the $\hbox {CD8}^{+}$ T cells which have been previously shown to infiltrate B16 murine melanoma, and the number of VSV particles. The model is parametrised using experimental data from Chen et al. (2011), Fernandez et al. (2002) (see also Fig. 2), and local sensitivity analysis is used to identify the parameters to which the tumour growth is most sensitive. We then use this model to investigate the anti-tumour effects of the oncolytic VSV, and how these effects interact with the anti-tumour immunity generated by the $\hbox {CD8}^{+}$ T cells and tumour-infiltrating macrophages. In particular, we aim to answer three main questions: (I) Can this mathematical model be used to test various hypotheses proposed in the experimental literature to reduce tumour burden? (II) How does macrophages plasticity affect the oncolytic virotherapies? (III) How does the balance between innate and adaptive immune responses impact the evolution of the tumour? We conclude in Sect. 4 with a summary of the results and a discussion on data availability and model complexity.

## Model Description

To investigate the effect of M1 and M2 macrophages on the anti-tumour oncolytic therapy with VSV and the interactions between macrophages and cytotoxic T cells, we consider a mathematical model that describes the time evolution of the following variables: the density of uninfected tumour cells ($x_{\text {u}}$), the density of virus-infected tumour cells ($x_{\text {i}}$), the density of virus particles ($x_{\text {v}}$), the density of M1 macrophages ($x_{\text {m1}}$), the density of M2 macrophages ($x_{\text {m2}}$) and the density of cytotoxic (effector) $\hbox {CD8}^{+}$ T cells ($x_{\text {e}}$); see also Fig. 1b. The time-evolution of these densities is described by the following equations:

1a $$\begin{aligned} \frac{dx_{\text {u}}}{dt}\,=\,&r x_{\text {u}}\left (1-\frac{x_{\text {u}}}{K} \right )-d_{v}x_{\text {v}}\frac{x_{\text {u}}}{h_{u}^{v}+x_{\text {u}}}-d_{u}x_{\text {u}}\frac{x_{\text {e}}}{h_{e}+x_{\text {e}}}- d_{m1}x_{\text {u}}\frac{x_{\text {m1}}}{h_{m}+x_{\text {m2}}} \\&+d_{m2}x_{\text {u}}\frac{x_{\text {m2}}}{h_{m}+x_{\text {m2}}}, \end{aligned}$$

1b $$\begin{aligned} \frac{dx_{\text {i}}}{dt}\,=\,&d_{v}x_{\text {v}}\frac{x_{\text {u}}}{h_{u}^{v}+x_{\text {u}}} -\delta _{i} x_{\text {i}}-d_{u}^{v}x_{\text {i}}\frac{x_{\text {e}}}{h_{e}+x_{\text {e}}} -d_{m1}^{v} x_{\text {i}}\frac{x_{\text {m1}}}{h_{m}+x_{\text {m2}}}, \end{aligned}$$

1c $$\begin{aligned} \frac{dx_{\text {v}}}{dt}\,=\,&H(t)+\delta _{i} b x_{\text {i}} -\omega x_{\text {v}} -d_{u}^{v}x_{\text {v}}\frac{x_{\text {e}}}{h_{e}+x_{\text {e}}} -d_{m1}^{v}x_{\text {v}}\frac{x_{\text {m1}}}{h_{m}+x_{\text {m2}}}, \end{aligned}$$

1d $$\begin{aligned} \frac{dx_{\text {m1}}}{dt}\,=\,& a_{1}^{v} (x_{\text {i}}+x_{\text {v}})+a_{1}^{u}x_{\text {u}}+p_{m1}x_{\text {m1}}\left (1-\frac{x_{\text {m1}}+x_{\text {m2}}}{M} \right )-x_{\text {m1}} \left(r_{m1}^{0}\right. \\& \left. + r_{m1}^{u}\frac{x_{\text {u}}}{h_{u}+x_{\text {u}}}\right) + x_{\text {m2}}\left ( r_{m2}^{0} +r_{m2}^{v}\frac{x_{\text {v}}}{h_{v}+x_{\text {v}}}\right ) - d_{em1}x_{\text {m1}}, \end{aligned}$$

1e $$\begin{aligned} \frac{dx_{\text {m2}}}{dt}\,=\,&a_{2}^{u}x_{\text {u}}+p_{m2}x_{\text {m2}}\left (1-\frac{x_{\text {m1}}+x_{\text {m2}}}{M} \right )+x_{\text {m1}}\left (r_{m1}^{0}+r_{m1}^{u}\frac{x_{\text {u}}}{h_{u}+x_{\text {u}}}\right) \\ & -x_{\text {m2}}\left ( r_{m2}^{0} +r_{m2}^{v}\frac{x_{\text {v}}}{h_{v}+x_{\text {v}}}\right ) -d_{em2}x_{\text {m2}}, \end{aligned}$$

1f $$\begin{aligned} \frac{dx_{\text {e}}}{dt}=&p_{e}\frac{x_{\text {m1}}}{h_{m}+x_{\text {m2}} }-d_{ee}x_{\text {e}}-d_{t}x_{\text {u}}x_{\text {e}}. \end{aligned}$$

These equations incorporate the following biological assumptions:

- The uninfected tumour cells, described by Eq. (1a), proliferate logistically with rate *r*, up to a carrying capacity *K*. Here, we assume a logistic growth because various experimental studies showed evidence of a reduced rate of tumour growth at larger sizes; see, for example, Laird (1964), Looney et al. (1980), Guiot et al. (2003). Further, we assume that the virus particles infect, at a rate $$d_{v}$$, only a certain proportion of the tumour (due to a multitude of obstacles associated with the tumour microenvironment  Wong et al. 2010). This can be modelled using a saturated term for the tumour–virus interactions, with $$h_{u}^{v}$$ the half saturation constant for tumour cells infected with the oncolytic virus particles. The uninfected tumour cells can be eliminated at a rate $$d_{u}$$ by IFN$$\gamma ^{+}$$$$\hbox {CD8}^{+}$$ T cells. Note that the saturated term in (1a) for tumour elimination by $$\hbox {CD8}^{+}$$ T cells describes the fact that only a fraction of these cells are IFN$$\gamma$$ positive—see also (Bridle et al. 2010). Moreover, we assume that the uninfected tumour cells can be eliminated by the M1 cells at a rate $$d_{m1}$$, since high numbers of infiltrating M1 macrophages are associated with good patient prognosis (Mantovani et al. 2006), and can eliminate mouse melanoma even in the absence of $$\hbox {CD8}^{+}$$ T cells (Hara et al. 1995). The presence of M2 cells inhibits the anti-tumour immune response generated by these M1 cells (Sica et al. 2008). Finally, we assume that these M2 cells support tumour growth at a rate $$d_{m2}$$, through the pro-tumour cytokines they secrete (Allavena and Mantovani 2012).
- The virus-infected tumour cells, described by Eq. (1b), die at a rate $$\delta _{i}$$ following viral replication and cell burst (see Eq. (1c)). The infected cells can be detected and eliminated at a rate $$d_{m1}^{v}$$($$\gg d_{m1}$$) by the M1 macrophages (Hashimoto et al. 2007; Italiani and Boraschi 2014), or at a rate $$d_{u}^{v}$$($$\gg d_{u}$$) by the IFN$$\gamma ^{+}$$$$\hbox {CD8}^{+}$$ T cells (Bridle et al. 2010). The anti-viral effect of M1 cells is inhibited by the presence of M2 cells.
- The virus, described by Eq. (1c), is injected into the system at some time $t>0$, and this virus administration is described by a function *H*(*t*) that is usually a combination of Heaviside functions; see caption of Fig. 2 for the description of *H*(*t*). The number of viral particles inside the tumour increases following the fast replication of these particles inside the tumour cells, causing the cells to burst open and release the particles. We denote by *b* the burst size, i.e., the number of viral particles released by one infected tumour cell. The half-life of these viral particles is $1/\omega$ (with non-immune human and mouse serum neutralising VSV very quickly  Tesfay et al. 2013). Moreover, the M1 macrophages can promote an anti-viral immune response, which leads to early clearance of virus particles at a rate $d_{m1}^{v}$(Ciavarra et al. 2005; Denton et al. 2016). This viral clearance can be suppressed by the M2 macrophages (Denton et al. 2016). An anti-viral immune response is triggered also by the viral antigen-specific $\hbox {CD8}^{+}$ T cells (Bridle et al. 2010; Christensen et al. 2004), which can reduce the level of virus particles at a rate $d_{u}^{v}$, e.g., through cytokine-mediated inhibition of viral replication (Komatsu et al. 1999; Christensen et al. 2004). We note that the rate at which the $\hbox {CD8}^{+}$T cells lyse the virus-infected cells could be different from the rate at which the virus particles are eliminated. For now we assume that both events are described by the same rate $d_{u}^{v}$. However, in Sect. 3.1 we will discuss also the possibility of having different $\hbox {CD8}^{+}$ T cells elimination rates for the virus-infected cells and for the virus particles.
- The M1 macrophages, described by Eq. (1d), are activated, at a rate $a_{m1}^{v}$, by viral pathogens and infected tumour cells that trigger the secretion of pro-inflammatory cytokines (such as IFN-$\gamma$) (Labonte et al. 2014). This immune response could also be activated, at a small rate $a_{1}^{u}$, by the uninfected tumour cells—if the macrophages could detect these tumour cells. The recruitment of M1 macrophages to the tumour site occurs at an average rate $p_{m1}$, up to a carrying capacity *M* (note that tissue-resident macrophages proliferate via a self-renewal process rather than through an influx of progenitors (Italiani and Boraschi 2014)). The M1$\rightarrow$M2 re-polarisation of macrophages occurs: (i) at a small constant rate $r_{m1}^{0}$ (due to cytokines, such as IL-4, IL-10, TGF-$\beta$, which can be produced by different types of healthy and immune cells), and (ii) at a tumour-dependent rate $r^{u}_{m1}x_{\text {u}}/(h_{u}+x_{\text {u}})$ (due to the anti-inflammatory cytokines produced by the tumour cells, e.g., TGB-$\beta$). The re-polarisation of $\hbox {M2}\rightarrow \hbox {M1}$ macrophages occurs at a small constant rate $r_{m2}^{0}$ (due to cytokines such as IFN-$\gamma$ or IL-12 produced by different types of cells in the environment). Recent experimental studies have shown that oncolytic viruses can be genetically modified to carry chemokines and cytokines that can induce a $\hbox {M2}\rightarrow \hbox {M1}$ re-polarisation (Guiducci et al. 2005). We denote by $r_{m2}^{v}$ this virus-induced re-polarisation rate, and for the beginning we consider $r_{m2}^{v}=0$; the case $r_{m2}^{v}>0$ will be discussed in Sect. 3.3. Finally, the M1 macrophages have a death rate of $d_{em1}$ (Yang et al. 2014; Italiani and Boraschi 2014).
- The M2 macrophages, described by Eq. (1e), are activated at a rate $a_{2}^{u}$ by cytokines such as IL-4, IL-10, IL-13, TGF-$\beta$, which are usually associated with a tumour-promoting environment (Labonte et al. 2014). These macrophages proliferate logistically at an average rate $p_{m2}$, up to their carrying capacity *M*. The M2$\leftrightarrow$M1 re-polarisation rates have been discussed in the previous paragraph. The M2 macrophages have a death rate of $1/d_{em2}$. Since many experimental studies on the turnover of macrophages do not distinguish between the M1 and M2 cells, throughout most of this study we will assume that $p_{m1}=p_{m2}:=p_{m}$, and $d_{em1}=d_{em2}:=d_{em}$. The cases where $d_{em1}\ne d_{em2}$ and $p_{m1}\ne p_{m2}$ will be investigated in Sect. 3.1, in the context of sensitivity analysis.
- The cytotoxic $\hbox {CD8}^{+}$ T cells, described by Eq. (1e), are activated and proliferate at a rate $p_{e}$ in the presence of tumour and viral antigens presented by M1 macrophages (Pozzi et al. 2005; Olazabal et al. 2008). (We acknowledge that both dendritic cells (DCs) and macrophages can prime naive $\hbox {CD8}^{+}$ T cells (Pozzi et al. 2005), with the DCs being considered the most potent antigen-presenting cells. However in this study we focus only on the macrophages since they are very abundant inside the tumour microenvironment, and thus they likely contribute to the initiation of T cell immunity. Moreover, the explicit inclusion of DCs in the model would only increase the complexity of the current system.) In contrast to other modelling studies on tumour–immune interactions following VSV therapy (see Macnamara and Eftimie 2015), here we assume that the tumour cells or virus particles do not influence directly the adaptive immune response, but they act through the innate response (M1 cells) which then activate the T cells. This is biologically realistic as experimental studies have shown that macrophage depletion suppressed the priming of $\hbox {CD8}^{+}$ T cells (Ciavarra et al. 2000). Finally, the $\hbox {CD8}^{+}$ T cells have a natural death rate $d_{e}$, and are inactivated by the tumour cells at a rate $d_{t}$.

### Remark 1

Many mathematical models in the literature assume linear interactions between different components of the system; see, for example, (Dingli et al. 2009; Kim et al. 2015; Eftimie and Hamam 2017; den Breems and Eftimie 2016). While these assumptions simplify the analysis of the models, they might not always be realistic since many biological interactions occur in a saturated-like manner and thus can be phenomenologically described by saturated functions of the form $\frac{x}{c+x}$ (where *x* is the variable under consideration, and *c* is a constant). As an example, experimental data in Dudley et al. (2002) showed that the percentage lysis of tumour cells by cytotoxic immune cells is saturated (with very large numbers of immune effector cells not leading to more effective tumour killing). Neither the tumour–virus interactions could always be described by linear interactions, since it would mean that larger numbers of oncolytic viruses would always kill the tumour. Experimental studies in Choi et al. (2018) with an oncolytic parapoxvirus showed that while virus levels of $10^{3}$, $10^{4}$ and $10^{5}$ PFU per tumour all lead to tumour reduction, the differences in tumour sizes were not always very significant— especially in later days (suggesting a sort of saturated effect). Regarding the VSV effects on B16F10 melanoma, the majority of studies in the literature focus on the percentage survival of mice. A different approach was shown in Bridle et al. (2009), where the authors investigated experimentally the effect of a fixed VSV level (i.e., $10^{7}$ PFU of VSV) on different tumour sizes, and showed that mice survival was almost the same when $1\times 10^{6}$ or $2\times 10^{6}$ B16F10 tumour cells were injected. Thus, in Eqs. (1a)–(1b) above we considered the term $x_{\text {v}}\frac{x_{\text {u}}}{h_{u}^{v}+x_{\text {u}}}$ to account for this saturated effect in tumour size during tumour–virus interactions. This form for the virus infection term is consistent with other studies that model oncolytic virotherapies; see (Wodarz and Komarova 2009; Komarova and Wodarz 2010).

### Parameter Approximation

To investigate the dynamics of tumour–immune–virus system (1), we first need to approximate the values of the parameters. In the following we discuss the approaches taken to identify the parameter values associated with tumour dynamics alone, the parameter values for the tumour–immune interactions, and the parameter values associated with the virus dynamics. All these values are summarised in Table 1.

Nevertheless, before we discuss these approaches, we need to emphasise that in mathematical and computational immunology, many researchers have used parameters already published in the literature: either measured experimentally following some particular experimental in vivo and/or in vitro studies, or parameters taken from other published mathematical and computational models (de Boer and Perelson 2013; Eftimie et al. 2016). This is a significant problem, since very few labs measure and estimate kinetics parameters, and even in this case the parameters are estimated for specific systems and might differ between studies (depending on the estimation method used, on the cell lines and virus strains used in the experiments, etc.) (de Boer and Perelson 2013; Eftimie et al. 2016). The only rigorous approach of dealing with this problem—which is both expensive and time consuming, but could lead to results that might have predictive power—is to estimate experimentally all parameters that appear in a model. In this study, we do not follow this rigorous approach, but rather follow the approaches taken by the majority of studies in the literature. To this end, we take some parameter values from the published immunological and mathematical literature (e.g., the in vitro proliferation rates of B16F10 murine cells (Danciu et al. 2013), the carrying capacity of macrophages (Eftimie and Hamam 2017), or the baseline M1$\leftrightarrow$M2 re-polarisation rates (Wang et al. 2012; Eftimie and Hamam 2017), while the rest of the parameters are approximated through model fitting to two different experiments with C57BL/6 mice (where we focus only on the control curves for tumour growth, and on the anti-tumour effect of wild-type VSV). Therefore, we do not expect that the results of this study will have predictive powers, but they will rather emphasise the range of various outcomes of tumour–virus–immune interactions.

**Table 1:** Summary of model parameters and their values used throughout this study

Param.	Value	Units	Description and references
*r*	0.924	$\text{{days}}^{-1}$	Proliferation rate for tumour cells (Danciu et al. 2013)
K	$3.3\times 10^{9}$	$\frac{{\text {cells}}}{{\text {vol}}}$	Carrying capacity for the tumour cells (Chen et al. 2011)
$d_{v}$	0.011	$\left (\frac{{\text {cells}}}{{\text {vol}}}\right )\times \left (\frac{{\text {PFU}}}{{\text {vol}}}\right )^{-1}\times ({{\text{days}}})^{-1}$	Infection rate of tumour cells with the oncolytic virus
$d_{u}$	0.44; 0.85	$\hbox {days}^{-1}$	Rate at which $\hbox {CD8}^{+}$ T cells eliminate uninfected tumour cells
$d_{u}^{v}$	4.4 ($\approx 0.85\times 5.17$)	$\hbox {days}^{-1}$	Rate at which $\hbox {CD8}^{+}$ T cells eliminate virus-infected tumour cells, as well as virus particles
$d_{m1}$	0.01; 0.29	$\hbox {days}^{-1}$	Rate at which M1 macrophages eliminate uninfected tumour cells
$d_{m1}^{v}$	1.5 ($\approx 0.29\times 5.17$)	$\hbox {days}^{-1}$	Rate at which M1 macrophages eliminate virus-infected tumour cells
$d_{m2}$	0.4	$\hbox {days}^{-1}$	Rate at which M2 macrophages support tumour growth
$h_{u}^{v}$	$10^{5}$	$\left (\frac{{\text {cells}}}{{\text {vol}}}\right )$	Half-saturation constant for the tumour cells infected with the oncolytic virus
$h_{m}$	$10^3$	$\left (\frac{{\text {cells}}}{{\text {vol}}}\right )$	Half-saturation constant for macrophages that support half the maximum immune response (leading to tumour elimination or tumour growth)
$h_{e}$	1.0	$\left (\frac{{\text {cells}}}{{\text {vol}}}\right )$	Half-saturation constant for the effector $\hbox {CD8}^{+}$ T cells that generate half the maximum cytotoxic immune response
$\delta _{i}$	0.475	$\hbox {days}^{-1}$	Rate at which the oncolytic virus kills an infected tumour cell (Zhu et al. 2009)
*b*	2500	$\left (\frac{{\text {PFU}}}{{\text {vol}}}\right )\,\times$ (cells)$^{-1}$(vol)	Number of VSV virus particles released from an infected cell, capable of forming plaques (Zhu et al. 2009)
$\omega$	2.0	$\hbox {days}^{-1}$	Death rate of oncolytic virus particles (Hwang and Schaffer 2013)
$a_{1}^{v}$	$1\times 10^{-6}$	$\hbox {days}^{-1}$	Activation rate of M1 macrophages in response to viral antigens
$a_{1}^{u}$	$3\times 10^{-6}$	$\hbox {days}^{-1}$	Activation rate of M1 macrophages in response to tumour antigens
$a_{2}^{u}$	$4\times 10^{-8}$	$\hbox {days}^{-1}$	Activation rate of M2 macrophages in response to tumour growth factors (TGF-$\beta$) or type-II cytokines in the tumour microenvironment
$p_{m1}$	0.22	$\hbox {days}^{-1}$	Proliferation rate of M1 cells
$p_{m2}$	0.22	$\hbox {days}^{-1}$	Proliferation rate of M2 cells
*M*	$10^{8}$	$\left (\frac{{\text {cells}}}{{\text {vol}}}\right )$	Carrying capacity of macrophages (Eftimie and Hamam 2017)
$r_{m1}^{0}$	$10^{-3}$	$\hbox {days}^{-1}$	Small baseline M1$\rightarrow$M2 re-polarisation rate in response to cytokines in the microenvironment (Wang et al. 2012; Eftimie and Hamam 2017)
$r_{m1}^{u}$	4.0	$\hbox {days}^{-1}$	M1$\rightarrow$M2 re-polarisation rate in response to tumour-supporting cytokines & growth factors
$r_{m2}^{v}$	0	$\hbox {days}^{-1}$	$\hbox {M2}\rightarrow \hbox {M1}$ re-polarisation rate in response to oncolytic viruses engineered to carry cytokines and chemokines that induce an M1-phenotype
$r_{m2}^{0}$	$10^{-3}$	$\hbox {days}^{-1}$	Small baseline $\hbox {M2}\rightarrow \hbox {M1}$ re-polarisation rate in response to cytokines in the microenvironment (Wang et al. 2012; Eftimie and Hamam 2017)
$h_{u}$	$5\times 10^{9}$	$\left (\frac{{\text {cells}}}{{\text {vol}}}\right )$	Half-saturation constant for the tumour cells that can trigger an M1$\rightarrow$M2 re-polarisation
$d_{em1}$	0.2	$\hbox {days}^{-1}$	Natural death rate of M1 macrophages (Yona et al. 2013)
$d_{em2}$	0.2	$\hbox {days}^{-1}$	Natural death rate of M2 macrophages (Yona et al. 2013)
$d_{ee}$	0.4	$\hbox {days}^{-1}$	Natural death rate of $\hbox {CD8}^{+}$ T cells (de Boer et al. 2003)
$p_{e}$	$2.07\times 10^3$	Cells/days	Activation of proliferation rate of $\hbox {CD8}^{+}$ T cells in the presence of M1 (pro-inflammatory) macrophages (de Boer et al. 2003)
$d_{t}$	$10^{-10}$	$\hbox {days}^{-1}$cells$^{-1}$	Inactivation rate of $\hbox {CD8}^{+}$ T cells by the tumour cells

- *Tumour dynamics alone* Experimental studies have shown that the murine B16 melanoma cells have a doubling time between 17.2 and 24 h (Danciu et al. 2013; Calvet et al. 2014). This corresponds to a proliferation rate of $r\in \left (\frac{\ln (2)}{(24/24)},\frac{\ln (2)}{(17.2/24)} \right )=(0.69,0.97)$/day. For the simulations we use an average value of $r=0.924$ corresponding to a doubling time of 18 h. Regarding the tumour carrying capacity *K* we focus on the data in Chen et al. (2011), where the maximum recorded tumour volume in the absence of any treatment was $\approx 3000mm^{3}$. Assuming that a volume of $1000mm^{3}$ contains approximately $10^{9}$ tumour cells (Friberg and Mattson 1997), we obtain a carrying capacity $K=3.0\times 10^{9}$ cells.
- *Immune response* During steady state conditions, circulating monocytes have a half life of 1–3 days (Yang et al. 2014), with some class of murine monocytes (Ly6C$^{-}$) exhibiting a longer steady-state half life of 5–7 days (Italiani and Boraschi 2014). Moreover, some macrophage populations can persist even longer, with the macrophages residing in intestinal lamina propria having a half-life of 3 weeks, and the alveolar macrophages persisting for years (Yona et al. 2013). Throughout this study we consider an average cell death rate of $d_{em}=0.2$/day (corresponding to a half life of 3.4 days). In regard to the macrophages carrying capacity, we take the approach in Eftimie and Hamam (2017) and assume that $M=10^{8}$. We also assume that the anti-viral immune response is much stronger than the anti-tumour immune response, and thus we choose $d_{u}^{v}=c_{0}d_{u}$, and $d_{m1}^{v}=c_{0}d_{m1}$, with $c_{0}\gg 1$. It is known that VSV is eliminated from the blood within 2–4 days after inoculation (Johnson et al. 2009). Therefore, we choose $c_{0}$ such that VSV particles persist in the system until day $t=17$ (i.e., 3–4 days after their second inoculation), above a detection level of at least 100 PFU/ml (Hodges et al. 2012). This leads to $c_{0}=5.17$ (so $d_{m1}^{v}=5.17\times d_{m1}$ and $d_{u}^{v}=5.17\times d_{u}$ for $t>10$ when the VSV is introduced in the system). In regard to the $\hbox {CD8}^{+}$ T cells, it is known that activated cells have a half-life of approximately 41 h (= 1.7 days), and a doubling time of about 8 h (= 0.3 days) (de Boer et al. 2003). This cell turnover translates into a decay rate $d_{ee}=0.4$/day, and an activation/proliferation rate $p_{e}=2.07\times 10^{3}$cells/day (assuming that there is an order of $10^{3}$ antigen-specific $\hbox {CD8}^{+}$ T cells per $\mu l$ of blood Bridle et al. 2009, 2010).
- *Virus dynamics* The burst size of the VSV varies between 50 plaque-forming units per cell (PFU/cell) to 8000 PFU/cell, with an average of 2500 PFU/cell (Zhu et al. 2009). Here we consider a baseline parameter value for the burst size of $b=2500$. Moreover, VSV-infected cells are lysed by the virus particles within 30–40 h post infection (Zhu et al. 2009). Assuming an average of 35 h, we thus consider $\delta _{i}=\ln (2.0)/35$h=0.47/day. Regarding the intracellular half-life of VSV particles, it has been shown in DePolo and Holland (1986) that it can vary between 5.3 and 12.5 h, depending on the viral mutant. Moreover, the extracellular half-life of retroviral vectors pseudotyped with VSV-G glycoprotein is between 3.5 and 8 h (Hwang and Schaffer 2013). In this study we assume a VSV half-life of 8 h, which corresponds to a baseline viral death rate $\omega =\ln (2.0)/(8/24)$/day=2.0/day.

To approximate the rest of the parameters associated with the anti-tumour immune response, we fit the model to two different B16F10 data sets from C57BL/6 mice, where we consider only the control data for tumour-growth curves (i.e., no additional treatments to boost up the anti-tumour or anti-viral immune responses). For the oncolytic virus, we focus only on the wild-type VSV (and its impact on the B16F10 cells).

- We first fit model (1) with no VSV ($x_{\text {v}}(t)=x_{\text {i}}(t)=0$) to the mean of (control) tumour data in Chen et al. (2011) (see Fig. 2a), since the injection of $5\times 10^{6}$ tumour cells into immunocompetent C57BL/6 mice likely triggers both innate and adaptive immune responses. Moreover a tumour doubling time of 18 h for B16F10 cells, as recorded in Danciu et al. (2013), Calvet et al. (2014), cannot explain the slow tumour growth in Chen et al. (2011)—see also Fig. 2a. The baseline tumour and immune parameter values ($d_{u}$, $h_{u}$, $h_{m}$, $h_{e}$, $d_{m1}$, $d_{m2}$, $a_{1}^{u}$, $a_{2}^{u}$, $p_{m}$, $r_{m1}^{0}$, $r_{m2}^{0}$, $r_{m1}^{u}$) obtained from this fitting are listed in Table 1. We must emphasise that given the very large parameter space, the values identified represent only one possible set of parameters that can fit the data. We also note that to be able to explain the M1:M2 ratios on days 7 and 14 (see Chen et al. 2011) we need to have $a_{1}^{u}\gg a_{2}^{u}$. Moreover, the M1$\rightarrow$M2 re-polarisation rate as a result of tumour growth ($r_{m1}^{u}$) needs to be much larger than the baseline re-polarisation rates ($r_{m1}^{0}$ and $r_{m2}^{0}$).
- Finally, we fit the full model (1) (with VSV) to the mean of B16F10 tumour growth data in Fernandez et al. (2002), where the mice received $2\times 10^{7}$ PFU of the wild-type VSV. The virus-related parameter values ($a_{1}^{v}$, $d_{v}$, $h_{u}^{v}$) that generated these baseline results are listed in Table 1. Note that these identified parameter values depend on our assumption that the $\hbox {CD8}^{+}$ T cells and M1 macrophages eliminate at the same rates the infected/uninfected tumour cells and the VSV particles. Since we had five free parameters ($a_{1}^{v}$, $d_{v}$, $h_{u}^{v}$, $d_{e}^{v}$, $d_{m1}^{v}$) that we could vary to fit the model (1) to the data, to obtain the best fit shown in Fig. 2 we had to incorporate also the assumption that the VSV injection (on day $t=10$) induces the release of tumour-associated antigens (TAAs), pathogen-associated molecular patterns (PAMPs) and danger-associated molecular patterns (DAMPs), which can activate macrophages within hours (Martin 2016; Melzer et al. 2017), and further lead to the activation of $\hbox {CD8}^{+}$ T cells within 4–7 days (following the release of tumour-associated antigens Diaz et al. 2007b; Melzer et al. 2017). Thus, we chose: $d_{m1}=0.01$ for $t<11$ and $d_{m1}=0.29$ for $t>11$, and $d_{u}=0.44$ for $t<15$, and $d_{u}=0.85$ for $t>15$). However, in the next section we will also discuss the situation when we assume that there are no different immune responses before/after VSV injection.

Finally, we note that the initial conditions for the in silico simulations performed throughout this study are summarised in Table 2.

**Table 2:** Summary of initial conditions used for the numerical simulations of system (1) throughout Sect. 3. These initial conditions aim to replicate the experimental conditions in Fernandez et al. (2002)

Variable	Description	Initial conditions
$x_{\text {u}}$	Density of uninfected tumour cells (cell numbers per volume) the day when the oncolytic virus is introduced	$x_{\text {u}}(0)=5\times 10^{5}$
$x_{\text {i}}$	Density of virus-infected tumour cells (cell numbers per volume)	$x_{\text {i}}(0)=0$
$x_{\text {v}}$	Density of virus particles (described as particles forming units (PFU) per volume)	$x_{\text {v}}(0)=0$
$x_{\text {m1}}$	Density of M1 macrophages (cell numbers per $\mu l$ of blood)	$x_{\text {m1}}(0)=0$
$x_{\text {m2}}$	Density of M2 macrophages (cell numbers per $\mu l$ of blood)	$x_{\text {m2}}(0)=0$
$x_{\text {e}}$	Density of $\hbox {CD8}^{+}$ T cells (cell numbers per $\mu l$ of blood)	$x_{\text {e}}(0)=0$

## Results

In the following we start the investigation into the role of macrophages on oncolytic virotherapies by performing first a local sensitivity analysis, to identify those parameters to which the model is most sensitive and to see which of these parameters are important in macrophages polarisation. Then we focus on a few virus-related and immune-related parameters (some identified as important during the sensitivity analysis), which will be varied to reproduce different experimental and clinical approaches aimed at controlling tumour growth. In this context, we will discuss the effect of varying these parameters on the size of M1 and M2 populations, and how this correlates with tumour control/elimination. This investigation will address mainly question (I) from the Introduction. To address questions (II) and (III) we will combine numerical simulations for transient system dynamics with steady-state analysis of long-term dynamics.

### Sensitivity Analysis

Before investigating the transient and long-term dynamics of model (1), we perform a local sensitivity analysis to obtain a first understanding on the importance of anti-tumour viral responses (i.e., virocentric perspective) versus anti-tumour immune responses (i.e., immunocentric perspective). Since we are interested in identifying the parameters that can slow down tumour relapse, we investigate the relative changes in tumour size on day $t=20$ [an arbitrarily-chosen day, when the tumour is growing back following the second VSV injection; see Fig. 2b(i)]. We compute this relative change as follows (Hamby 1994; Olufsen and Ottesen 2013):

$${{\frac{{\Delta x_{{\text{u}}} }}{{x_{{\text{u}}} }}} \mathord{\left/ {\vphantom {{\frac{{\Delta x_{{\text{u}}} }}{{x_{{\text{u}}} }}} {{{\left| {\frac{{\Delta param}}{{param}}} \right| = \frac{{x_{{\text{u}}}^{{old}} (20) - x_{{\text{u}}}^{{new}} (20)}}{{x_{{\text{u}}}^{{old}} (20}}} \mathord{\left/ {\vphantom {{\left| {\frac{{\Delta param}}{{param}}} \right| = \frac{{x_{{\text{u}}}^{{old}} (20) - x_{{\text{u}}}^{{new}} (20)}}{{x_{{\text{u}}}^{{old}} (20}}} {\left| {\frac{{param^{{old}} - param^{{new}} }}{{param^{{old}} }}} \right|}}} \right. \kern-\nulldelimiterspace} {\left| {\frac{{param^{{old}} - param^{{new}} }}{{param^{{old}} }}} \right|}},}}} \right. \kern-\nulldelimiterspace} {{{\left| {\frac{{\Delta param}}{{param}}} \right| = \frac{{x_{{\text{u}}}^{{old}} (20) - x_{{\text{u}}}^{{new}} (20)}}{{x_{{\text{u}}}^{{old}} (20}}} \mathord{\left/ {\vphantom {{\left| {\frac{{\Delta param}}{{param}}} \right| = \frac{{x_{{\text{u}}}^{{old}} (20) - x_{{\text{u}}}^{{new}} (20)}}{{x_{{\text{u}}}^{{old}} (20}}} {\left| {\frac{{param^{{old}} - param^{{new}} }}{{param^{{old}} }}} \right|}}} \right. \kern-\nulldelimiterspace} {\left| {\frac{{param^{{old}} - param^{{new}} }}{{param^{{old}} }}} \right|}},}}{\text{ }}$$

where $param^{new}=param^{old}\pm\, 80\% param^{old}$. We chose to vary the baseline parameters by $\pm\, 80\%$ as we aim to explore large parameter fluctuations around these baseline values, which are also proportional to the magnitudes of the parameters identified in Table 1.

**Fig. 3:**
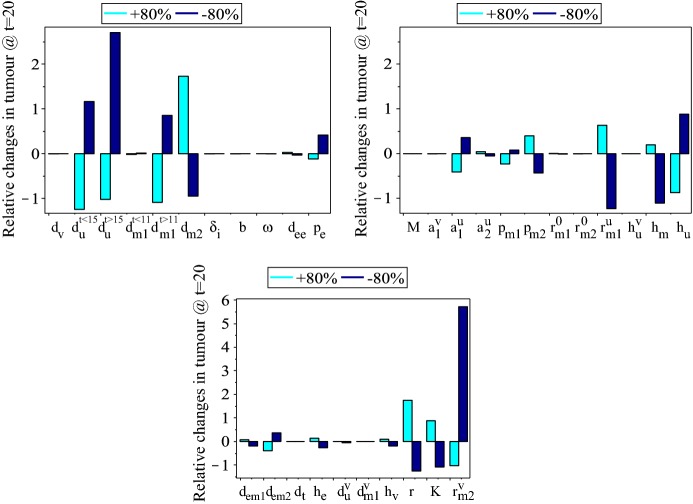
Relative sensitivity of tumour size on day $t=20$ to $\pm\, 80\%$ changes in the baseline parameter values listed in Table 1. We denoted by $d_{m1}^{t<11}$ and $d_{u}^{t<15}$ the values of $d_{m1}$ and $d_{u}$ before the M1 and $\hbox {CD8}^{+}$ T cells response to the release of TAAs/PAMPs/DAMPs by the virus-lysed tumour cells. Also, we denoted by $d_{m1}^{t>11}$ and $d_{u}^{t>15}$ the values of $d_{m1}$ and $d_{u}$ after the increase in the M1 and $\hbox {CD8}^{+}$ T cells response to the release of TAAs/PAMPs/DAMPs. For $r_{m2}^{v}$ we started with an arbitrary value of $r_{m2}^{v}=0.5$ (much smaller than the baseline value of $r_{m1}^{u}$), which we then varied by $\pm 80\%$

Figure 3 shows that tumour relapse (on day $t=20$) is most sensitive to the virus-induced $\hbox {M2}\rightarrow \hbox {M1}$ re-polarisation ($r_{m2}^{v}$), as well as to the anti-tumour $\hbox {CD8}^{+}$ T cell immune response ($d_{u}^{t<15}$,$d_{u}^{t>15}$), the anti-tumour M1 immune responses following the lysis of tumour cells by the VSV particles and the release of TAAs/PAMPs/DAMPs ($d_{m1}^{>11}$), the half-saturation density of M2 cells that inhibit the anti-tumour M1 responses ($h_{m}$), the pro-tumour M2 responses ($d_{m2}$), and the tumour-induced M1$\leftrightarrow$M2 re-polarisation rate ($r_{m1}^{u}$) combined with the half-saturation density of tumour cells that trigger the M1$\leftrightarrow$M2 re-polarisation ($h_{u}$). Other parameters that can impact significantly tumour relapse are: the tumour proliferation rate (*r*), the tumour carrying capacity (*K*), the activation rate of M1 cells ($a_{1}^{u}$), the proliferation rate of *M*2 cells ($p_{m2}$).

Note that with the exception of $r_{m2}^{v}$, which will be discussed in more detail in Sect. 3.3, the model does not seem to be particularly sensitive to the majority of virus-related parameters, even when some of these parameters are quite large; e.g., see *b*, $\delta _{i}$ in Table 1. This suggests that the virus alone might not have much impact on tumour control/elimination. Rather these outcomes are the results of the combined effect between viruses and anti-tumour immune responses, represented here by the $\hbox {CD8}^{+}$ T cells and M1 cells. Moreover, the fact that the tumour-induced macrophages re-polarisation rate $r_{m1}^{u}$ has such a big impact on tumour decay suggests that any oncolytic virotherapies should take into consideration also the effect of tumour environment on macrophages polarisation.

Overall, these sensitivity results emphasise the importance of both innate and adaptive immunity on tumour relapse, thus supporting the immunocentric point of view (Alemany and Cascallo 2009). Another aspect emphasised by these sensitivity results is that even small changes in these immune-related parameters (due to environmental heterogeneity and stochasticity Satija and Shalek 2014; Jiménez-Sánchez et al. 2017; Papalexi and Satija 2018) could affect significantly the outcome of the oncolytic viral therapy and the survival of the patient.

#### Remark 2

We have also investigated (not shown here) tumour sensitivity to the rate at which the $\hbox {CD8}^{+}$ T cells lyse the virus particles, which we separated from the rate at which the $\hbox {CD8}^{+}$ T cells lyse the virus-infected cells. The sensitivity results did not show any significant difference compared to the case when these two rates are the same (see tumour sensitivity to $d_{u}^{v}$, in the bottom panel of Fig. 3).

In the following we use model (1) to investigate different immunological hypotheses associated with changes in the parameters to which the tumour is most sensitive.

### Baseline Model Dynamics

**Fig. 4:**
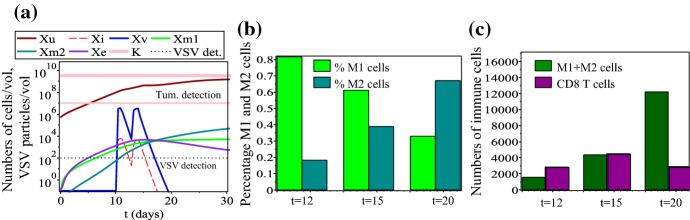
Baseline dynamics of model (1): **a** Overall model dynamics; The thin horizontal line depicts the tumour detection threshold which, according to Friberg and Mattson (1997) is about $10^{7}$ cells. The thin dotted line depicts VSV detection level which, according to Hodges et al. (2012) is about $10^{2}$ cells. **b** The percentages of M1 and M2 cells on three different days: $t=12$, $t=15$ and $t=20$. **c** The total numbers of macrophages and $\hbox {CD8}^{+}$ T cells on three different days: $t=12$, $t=15$ and $t=20$

We start the investigation into the dynamics of model (1) by showing in Fig. 4(a) the baseline dynamics when the VSV is injected on days $t=10$ and $t=13$ [as in Fernandez et al. (2002)]. In this case, the presence of the oncolytic virus that is detected for up to 4 days after the last injection (assuming that detection threshold is $~10^{2}\hbox {PFU/ml}$ Hodges et al. 2012), leads to a slow-down in tumour growth. This slow-down in tumour growth is also the result of a large $\hbox {CD8}^{+}$ T cell population (Fig. 4(c)) and a large M1:M2 ratio (Fig. 4(b)). Tumour relapse is associated with both a small M1:M2 ratio and a large M1+M2 population, as well as a small $\hbox {CD8}^{+}$ T cell population.

Next, we move away from the experimental studies in Fernandez et al. (2002), Chen et al. (2011) and investigate whether model (1) can reproduce various anti-tumour treatment approaches proposed by different experimental studies. The parameters that we will vary in this context, which can be controlled experimentally, will help us further propose hypotheses regarding the best approaches for tumour control/elimination.

### Changes in Virus-Related and Immune-Related Parameters

**Fig. 5:**
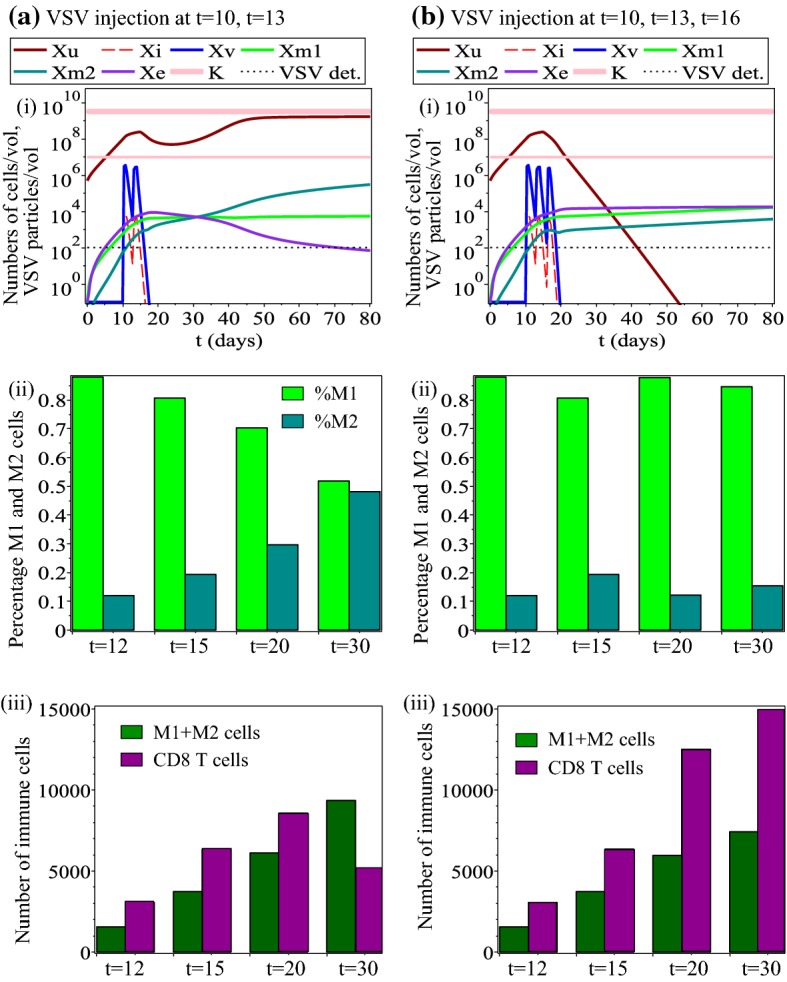
Dynamics of model (1) when we assume that the oncolytic virus can induce a $\hbox {M2}\rightarrow \hbox {M1}$ re-polarisation: $r_{m2}^{v}=0.5$. **a** We consider two VSV injections of $2\times 10^{7}$PFU on days $t=10$ and $t=13$; **b** We consider three VSV injections of $2\times 10^{7}$PFU on days $t=10$, $t=13$ and $t=16$. Sub-panels (i) show the tumour-immune-virus dynamics, sub-panels (ii) show the percentages of M1 and M2 cells on days $t=12$, $t=15$, $t=20$ and $t=30$, and sub-panels (iii) show the total number of M1+M2 macrophages and total numbers of $\hbox {CD8}^{+}$ T cells on days $t=12$, $t=15$, $t=20$, $t=30$

*Changes in virus-induced macrophages re-polarisation rate*$r_{m2}^{v}$. Since the sensitivity analysis showed that one of the parameters that have the largest impact on tumour dynamics is $r_{m2}^{v}$, next we investigate the assumption that the oncolytic virus is engineered to induce a $\hbox {M2}\rightarrow \hbox {M1}$ re-polarisation (Guiducci et al. 2005; Masemann et al. 2018). We need to emphasise that such a $\hbox {M2}\rightarrow \hbox {M1}$ macrophage re-polarisation has been obtained experimentally with oncolytic adenoviruses (Guiducci et al. 2005) or oncolytic influenza viruses (Masemann et al. 2018). A very recent study (McCanless 2019) also showed that a VSV strain can induce a $\hbox {M2}\rightarrow \hbox {M1}$ re-polarisation in the context of breast cancer, as determined by the increased secretion of TNF-$\alpha$—a cytokine associated with M1 responses, and also with $\hbox {CD8}^{+}$ T cell responses (Bertrand et al. 2016). To investigate computationally the effect of increasing the virus-induced $\hbox {M2}\rightarrow \hbox {M1}$ re-polarisation rate in the context of B16F10 melanoma, next we assume that $r_{m2}^{v}=0.5$. Figure 5 shows the dynamics of model (1) when we consider multiple VSV treatments: (a) two VSV treatments (as in Fernandez et al. (2002)), (b) three VSV treatments. First, we note that tumour reduction and eventual elimination is always associated with a large percentage of M1 macrophages (while tumour relapse is associated with an increased percentage of M2 macrophages). Second, we note that a third round of VSV injection could lead to a persistent $\hbox {CD8}^{+}$ T cells response (panel (b)(iii)), which eventually causes tumour elimination; this response is consistent with previous experimental observations on the activation of $\hbox {CD8}^{+}$ cells following VSV delivery (Bridle et al. 2009). Note that in this case, the total number of tumour-infiltrating macrophages does not seem to play a major role in tumour elimination or persistence—only the ratio M1:M2 does. Therefore, we can speculate that offering multiple rounds of oncolytic therapies with viruses aimed at re-polarising the macrophages towards a M1 phenotype (e.g., GM-CSF-armed viruses; Deng et al. 2016), which leads to an increase in the $r_{m2}^{v}$ rate, could improve current oncolytic and immune cancer therapies.

**Fig. 6:**
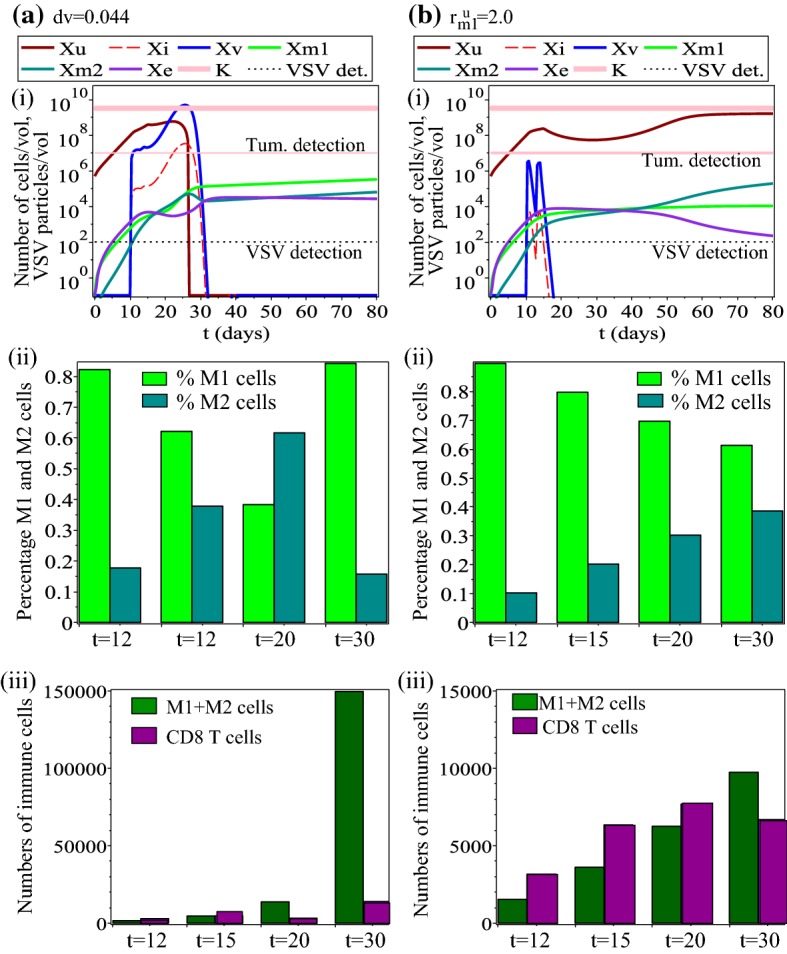
Dynamics of model (1) as we vary: **a** a virus-related parameter ($d_{v}$); **b** an immune-related parameter ($r_{m1}^{u}$). Sub-panels (i) show the whole dynamics of the system. Sub-panels (ii) show the percentages of M1 and M2 cells on four days during and after VSV treatment: $t=12$, $t=15$, $t=20$, $t=30$. Sub-panels (iii) show the total size of the macrophages and $\hbox {CD8}^{+}$ T cell populations on four days during and after VSV treatment: $t=12$, $t=15$, $t=20$, $t=30$

*Changes in virus-related parameters* Next, we investigate the dynamics of model (1) as we vary the main virus-related parameter ($d_{v}$) to which the tumour is not very sensitive (see Fig. 3), but which is important in the context of anti-tumour treatments as many experimental studies emphasise the importance of improving the delivery of these viruses into the tumour cells (Vähä-Koskela and Hinkkanen 2014). Figure 6(a) shows that a significant increase (i.e., by a factor of 4) in the rate $d_{v}$ at which the oncolytic virus infects the tumour cells can lead to tumour control (for $t\in (15,25)$) and even tumour elimination (for $t>27$). This tumour clearance is the result of a very large viral infection (see the curves for $x_{\text {v}}$ and $x_{\text {i}}$ in panel (a)(i)), which is then followed by a large immune response. Note that the immune response following tumour clearance is characterised by a very large number of macrophages (M1+ M2), with a large M1:M2 ratio. It can be easily checked that similar virus-induced tumour elimination can be obtained if we decrease (e.g., by a factor of 4) the killing rates of virus particles by the $\hbox {CD8}^{+}$ T cells ($d_{e}^{v}$) and M1 cells ($d_{m1}^{v}$), to simulate a reduction in the anti-viral immune response as suggested by many experimental and clinical studies; e.g., via immunosupressive chemotherapeutics such as cyclophosphamide, which affects both $\hbox {CD8}^{+}$ T cell and macrophage populations (Filley and Dey 2017; Santosuosso et al. 2002; Hanoteau et al. 2017). Therefore, we can speculate that by increasing the rate at which the oncolytic virus infects the tumour cells (e.g., by focusing on the reduction of physical barriers inside the tumour, which allows for better virus spread (Alzahrani et al. 2019; Vähä-Koskela and Hinkkanen 2014), or by focusing on the reduction of anti-viral immune responses), we could also trigger large sub-sequent anti-tumour innate and adaptive immune responses that might lead to permanent tumour elimination.

*Changes in tumour-induced macrophages re-polarisation rate*$r_{m1}^{u}$. Over the past few years, various experimental and clinical studies focused on preventing the differentiation of M2 macrophages for better anti-tumour outcomes (e.g., by depriving the tumour of growth factors and thus preventing the M1$\rightarrow$M2 differentiation; see the review in Heusinkveld and van der Burg (2011) or the clinical study in Coward et al. (2011)). To investigate this treatment approach, next we study computationally the effect of decreasing parameter $r_{m1}^{u}$ which controls this tumour-induced M1$\rightarrow$M2 differentiation. Figure 6b shows that a significant decrease in the re-polarisation rate $r_{m1}^{u}$ can lead to tumour control (and even elimination; e.g. for $r_{m1}^{u}=1$, not shown here). Here, tumour control is the result of a relatively large $\hbox {CD8}^{+}$ T cell population, which seems to dominate the immune response for $t\in (15,25)$, and a very large M1:M2 ratio (although the macrophages population as a whole, M1+M2, is quite low). The VSV level is relatively low, as the anti-tumour and anti-viral immune responses control the dynamics of the system. These numerical results are consistent with some experimental studies showing the activation of $\hbox {CD8}^{+}$ T cells following the administration of VSV (Bridle et al. 2009). However, the tumour relapse triggered by the increase in the total number of macrophages (as well as the M2:M1 ratio), suggests the necessity of tracking experimentally not only the T cells’ response to VSV [as done in Bridle et al. (2009)], but also the interactions between T cells and macrophages.

Overall, Figs. 5 and 6 suggest that there are two mechanisms through which the tumour is kept under control and even eliminated: a viral-dominated response (as in Fig. 6a) and an immune-dominated response (as in Fig.  5b). We need to emphasise that the viral-dominated anti-tumour response generates quickly a very strong immune response that ensures persistent tumour elimination. This kind of dynamics might lead to a confusion as to whether the tumour is eliminated by the immune response or by the viral replication. We stress that these two mechanisms (virocentric vs. immunocentric) depend on the model parameters, which can be different for different cells inside the same patient (as shown by a recent clinical study on the heterogeneity of tumour–immune microenvironments; see Jiménez-Sánchez et al. 2017).

**Fig. 7:**
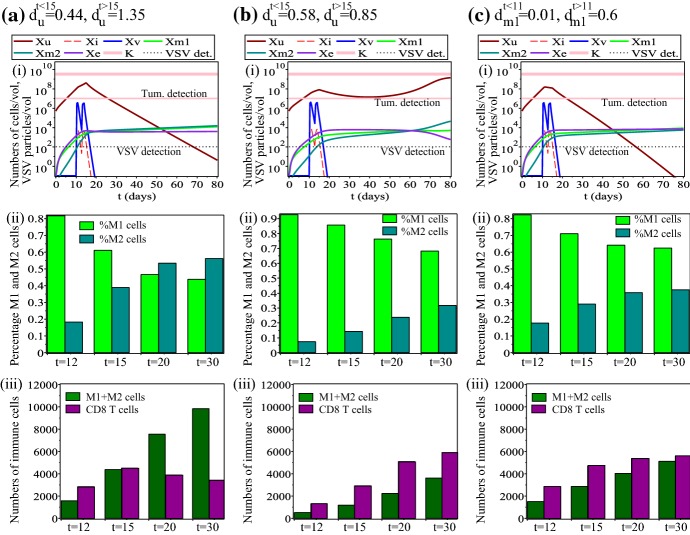
Dynamics of model (1) as we increase the following parameters: **a**$d_{u}^{t>15}=1.35$; **b**$d_{u}^{t<15}=0.58$; **c**$d_{m1}^{t>11}=0.6$. All other parameters are as in Table 1. Sub-panels (i) show the whole dynamics of the system; sub-panels (ii) show the percentages of M1 and M2 cells on days $t=12$, $t=15$, $t=20$, $t=30$; sub-panels (iii) show the total size of the macrophage (M1+M2) and $\hbox {CD8}^{+}$ T cell populations on days $t=12$, $t=15$, $t=20$, $t=30$

*Changes in anti-tumour immunity rates* Finally, we investigate the effect of shifting the immune response from viral antigens to tumour antigens, for example, by injecting oncolytic viruses that carry TAAs (Bridle et al. 2010; Melcher et al. 2011). In our model, this is described by the increase the tumour killing rates of $\hbox {CD8}^{+}$ T cells ($d_{u}$) and M1 cells ($d_{m1}$) before/after the release of TAAs/PAMPs/DAMPs from the lysed tumour cells. Figure 7 shows three types of immune responses that can lead to tumour control/elimination: (a) an immune response characterised by relatively low M1:M2 ratios, a very large number of tumour-infiltrating macrophages (M1+M2), and a relatively small number ($<3500$ at $t=30$) of $\hbox {CD8}^{+}$ T cells; (b) an immune response characterised by high M1:M2 ratios, a small number of tumour-infiltrating macrophages (M1+M2), and a high number ($>5800$ at $t=30$) of $\hbox {CD8}^{+}$ T cells; (c) an immune response characterised by relatively high M1:M2 ratios, a small number of tumour-infiltrating macrophages (M1+M2), and a median number ($\approx 5500$ at $t=30$) of $\hbox {CD8}^{+}$ T cells. The most puzzling outcome is the one shown in panels (a), since one would expect to see tumour relapse when M2>M1. We suspect that the relatively similar numbers of M1 and M2 cells (the M2 population is only slightly larger than the M1 population), together with the very large $d_{u}^{t>15}$ value describing a very strong anti-tumour $\hbox {CD8}^{+}$ T cell response despite low $\hbox {CD8}^{+}$ T cell numbers, play an important role in controlling tumour growth/elimination. This result is consistent with the outcome of various experimental approaches focused on priming of $\hbox {CD8}^{+}$ T cells with oncolytic viruses that carry TAAs (Diaz et al. 2007a; Bridle et al. 2009, 2010), which showed enhanced anti-tumoural activities via increased IFN-$\gamma$ production and not via increased T cell numbers (Diaz et al. 2007a). However, this increased anti-tumoural activity is still difficult to control in cancer patients, and is the subject of continuous research.

We note here that varying other immune parameters to which the tumour is sensitive—see Fig. 3—leads to a model dynamics similar to the one shown in Fig. 7. Moreover, we believe that using other sets of parameters that could fit the data in Chen et al. (2011) and Fernandez et al. (2002) would likely lead to tumour-immune-virus dynamics similar to the cases shown in Figs. 5, 6 and 7.

**Fig. 8:**
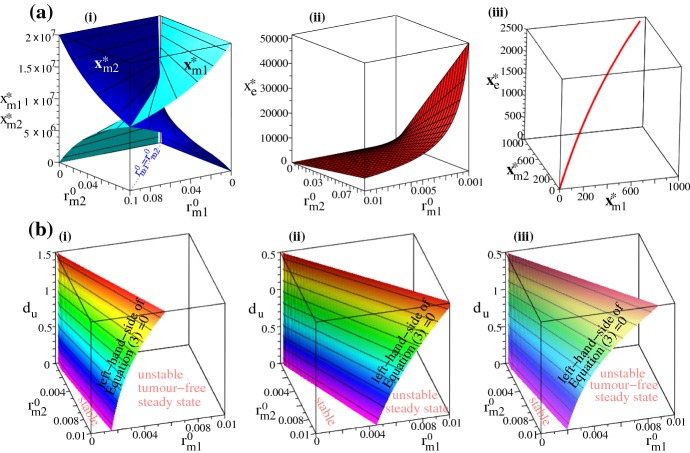
**a** Tumour-free steady state ($0,0,0,x_{\text {m1}}^{*},x_{\text {m2}}^{*},x_{\text {e}}^{*}$); Sub-panels (i)–(ii) show the immune states (versus $r_{m1}^{0}$ and $r_{m2}^{0}$) as given by equations (2) (where the very low $x_{\text {e}}^{*}$ value is the result of $h_{m}=1000$— see Eq. (2a)); the red curve in sub-panel (iii) describes the intersection of the surfaces defining the three immune states in the ($x_{\text {m1}}^{*},x_{\text {m2}}^{*},x_{e}^{*}$) space; here we fix $r_{m1}^{0}=r_{m2}^{0}=0.001$, as in Table 1. **b** Stability regions for the tumour-free steady state, in the parameter space given by ($r_{m1}^{0},r_{m2}^{0},d_{u}$), as we vary the anti-tumour/pro-tumour immune responses. The surface describes the left-hand-side of (3), when it becomes equal to zero, and thus separates the stability and instability regions. (i) baseline $d_{m1}$ and $d_{m2}$ values (as in Table 1); (ii) $d_{m1}=0.6$, $d_{m2}=0.4$; (iii) $d_{m1}=0.2$, $d_{m2}=0.1$. All other parameter values are as in Table 1

### Long-term Dynamics: Steady States and Their Stability

The numerical simulations presented in the previous sections suggested the possibility of having long-term behaviours characterised by tumour elimination and the persistence of an immune response, or behaviours characterised by the coexistence of tumour and immune cells. In the following we aim to obtain a better understanding of these behaviours by focusing on the steady states $(x_{\text {u}}^{*},x_{\text {i}}^{*},x_{\text {v}}^{*},x_{\text {m1}}^{*},x_{\text {m2}}^{*},x_{\text {e}}^{*})$ exhibited by model (1). To this end, we focus only on the following three cases: (i) tumour-absent states (that can shed slight on the combinations of parameters that can lead to tumour elimination); (ii) tumour-present virus-absent states (that can shed light on the combinations of parameters important in anti-tumour immunity); and (iii) tumour-present, virus-present and immune-present states (that can shed light on the combinations of parameters important in anti-tumour and anti-viral immunity, as well as oncolytic activities).

**Fig. 9:**
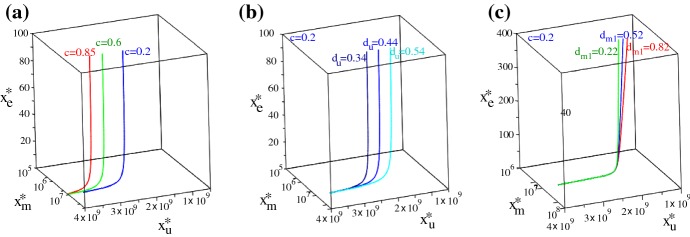
Steady states ($x_{\text {u}}^{*},0,0,x_{\text {m1}}^{*},x_{\text {m2}}^{*},x_{\text {e}}^{*}$) as given by the intersection of the surfaces (4a) and (4b), for three different values of: **a** the approximating parameter *c*: $c=0.2$, $c=0.6$ and $c=0.85$; **b** we fix $c=0.2$ and vary the elimination rate $d_{u}$ of tumour by $\hbox {CD8}^{+}$ T cells, from $d_{u}=0.34\rightarrow d_{u}=0.54$; **c** we fix $c=0.2$ and vary the elimination rate $d_{m1}$ of tumour by M1 cells, from $d_{m1}=0.22\rightarrow d_{m1}=0.82$. Note that here we graph the total macrophage population $x_{\text {m}}^{*}=x_{\text {m1}}^{*}+x_{\text {m2}}^{*}$, and the variations in *c*, $d_{u}$ and $d_{m1}$ do not seem to affect significantly this total $x_{\text {m}}^{*}$ population, only the $x_{\text {u}}^{*}$ population.

**Fig. 10:**
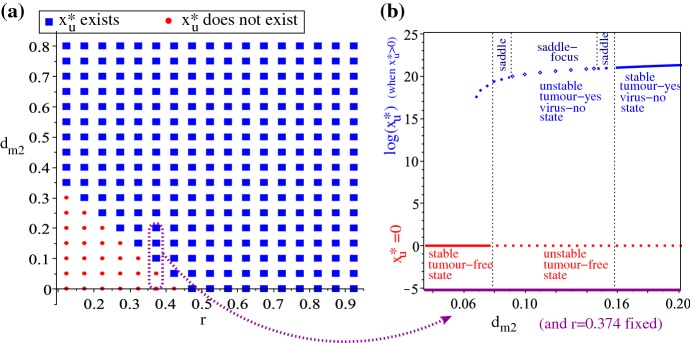
**a** Diagram in the ($r,d_{m2}$)-space showing the existence of the tumour-present virus-absent state ($x_{\text {u}}^{*},0,0,x_{\text {m1}}^{*},x_{\text {m2}}^{*},x_{\text {e}}^{*}$), as given by Eq. (5). **b** Bifurcation diagram showing the existence and stability of the tumour-free state ($0,0,0,x_{\text {m1}}^{*},x_{\text {m2}}^{*},x_{\text {e}}^{*}$) and the tumour-present virus-absent state ($x_{\text {u}}^{*},0,0,x_{\text {m1}}^{*},x_{\text {m2}}^{*},x_{\text {e}}^{*}$), as we vary the parameter $d_{m2}$ in Equation (5) over the range (0.03, 0.2) while fixing $r=0.374$; this parameter range is indicated also in **a**, where we see a transition between existence and non-existence of the tumour-present virus-absent $x_{\text {u}}^{*}$ state.

**Fig. 11:**
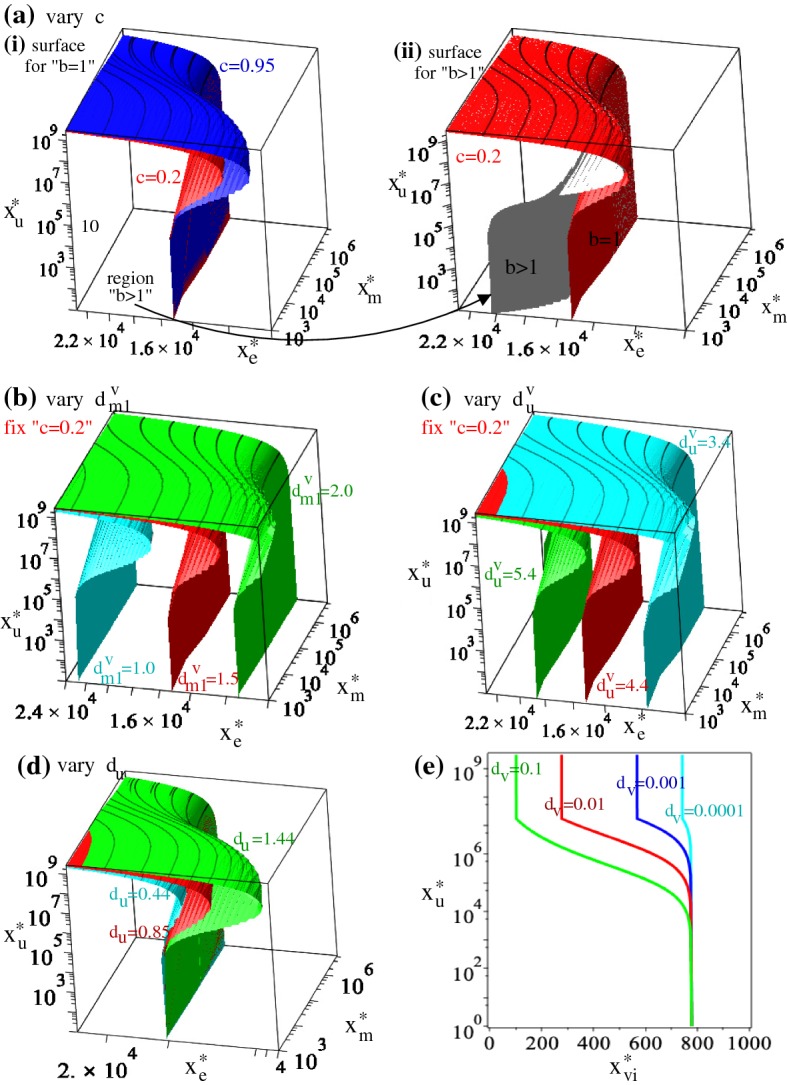
Steady state ($x_{\text {u}}^{*},x_{\text {i}}^{*},x_{\text {v}}^{*},x_{\text {m1}}^{*},x_{\text {m2}}^{*},x_{\text {e}}^{*}$) as given by Eqs. (6)–(6), when we vary: **a** parameter $c=x_{\text {m2}}^{*}/(h_{m}+x_{\text {m2}}^{*})\in (0.1)$ which gives some information about the density of M2 cells (and implicitly the density of M1 cells, as $x_{\text {m1}}^{*}=x_{\text {m}}^{*}-x_{\text {m2}}^{*}$. We focus on two cases: (i) $b=1$, and (ii) $b>1$ (with $C_{0}=6\times 10^{6}$ in (6)); **b** the rate $d_{m1}^{v}$ at which the M1 cells eliminate the virus and virus-infected cells; **c** the rate $d_{u}^{v}$ at which the $\hbox {CD8}^{+}$T cells eliminate the virus and virus-infected cells; **d** the rate $d_{v}$ at which the virus infects the tumour cells. The rest of parameters are as in Table 1

(i) *Tumour-absent states* Since the previous numerical results (e.g., Fig. 6b) showed that tumour could be eliminated, in the following we investigate the conditions on various parameters that ensure the existence of tumour-free steady states and their stability. To this end, we focus on the steady states that have $x_{\text {u}}=0$. Using Eqs. (1b)–(1c) this implies that $x_{\text {i}}=0$ and $x_{\text {v}}=0$. The steady-state values for the immune cells are given by (see also Appendix A):
  2a $$\begin{aligned}&x_{\text {m1}}^{*}=\frac{x_{\text {m}}^{*}r_{m2}^{0}}{r_{m1}^{0}+r_{m2}^{0}}, \; x_{\text {m2}}^{*}=\frac{x_{\text {m}}^{*}r_{m1}^{0}}{r_{m1}^{0}+r_{m2}^{0}}, \; x_{\text {e}}^{*}=\frac{p_{e}}{d_{ee}}\frac{x_{\text {m}}^{*}r_{m2}^{0}}{(r_{m1}^{0}+r_{m2}^{0})h_{m}+x_{\text {m}}^{*}r_{m1}^{0},} \end{aligned}$$
  where
  2b $$\begin{aligned}&\;\;\; x_{\text {m}}^{*}=\frac{(p_{m}-d_{em})M}{p_{m}}>0, \;\;\; \text {and} \\&\qquad p_{m}:=p_{m1}=p_{m2}, \;\;\; d_{em}:=d_{em1}=d_{em2}. \end{aligned}$$
  Here, $x_{\text {m}}^{*}=x_{\text {m1}}^{*}+x_{\text {m2}}^{*}$, the total macrophage population at steady state. It is possible to have also $x^{*}_{\text {m1}}=x^{*}_{\text {m2}}=x^{*}_{\text {e}}=0$, but this state is always unstable and we will not investigate it any further. Note that these immune-present tumour-free states (2) depend on the baseline re-polarisation rates $r_{m2}^{0}$ and $r_{m1}^{0}$, as well as on the proliferation and death rates of macrophages and $\hbox {CD8}^{+}$ T cells. In Fig. 8a(i)–(ii) we graph these states in the ($r_{m1}^{0},r_{m2}^{0}$)-space—mainly to see. We observe that the tumour-free state can be characterised by $x_{\text {m1}}^{*}>x_{\text {m2}}^{*}$ (for $r_{m2}^{0}>r_{m1}^{0}$) or by $x_{\text {m1}}^{*}<x_{\text {m2}}^{*}$ (for $r_{m1}^{0}>r_{m2}^{0}$). The tumour-free steady state $(0,0,0,x_{\text {m1}}^{*},x_{\text {m2}}^{*},x_{\text {e}}^{*})$, with $x_{\text {m1}}^{*}$, $x_{\text {m2}}^{*}$ and $x_{\text {e}}$ given by Eq. (2) is asymptotically stable provided that the parameters listed in Table 1 satisfy the following inequality:
  3 $$\begin{aligned} r-d_{u}\frac{x_{\text {e}}^{*}}{h_{e}+x_{\text {e}}^{*}}-d_{m1}\frac{x^{*}_{\text {m1}}}{h_{m}+x^{*}_{\text {m2}}}+d_{m2}\frac{x^{*}_{\text {m2}}}{h_{m}+x^{*}_{\text {m2}}}<0. \end{aligned}$$
  The proof is given in Appendix A. The surface described by the left-hand-side of inequality (3) is sketched in Fig. 8b. More precisely, in Fig. 8b(i) we sketch the expression that appears in the left-hand-side of the inequality (3) for the baseline parameter values shown in Table 1, while in Fig. 8b(ii),(iii) we sketch it for different $d_{m1}$ and $d_{m2}$ values. Note that the stability region increases over the ($r_{m1}^{0},r_{m2}^{0}$)-space for large $d_{u}$ and $d_{m1}$, as well as for small $d_{m2}$.
(ii) *Tumour-present virus-absent states* Since the previous numerical simulations (see Figs. 4, 5, 6 and 7) showed that the virus is always eliminated (which is consistent with experimental studies discussing VSV neutralisation by non-immune human and mouse serum; see Tesfay et al. 2013), next we focus on the steady states ($x_{\text {u}}^{*},0,0,x_{\text {m1}}^{*},x_{\text {m2}}^{*},x_{\text {e}}^{*}$). These states are given implicitly by the following two equations, which were obtained by combining the steady-state Equations (1a) and (1f) (where, for simplicity, we assumed in (1a) that $d_{m2}\times x_{\text {m2}}^{*}/(h_{m}+x_{\text {m2}}^{*})\approx d_{m2}\times c$ with $c\in (0,1)$), and by adding the steady-state Equations (1d) + (1e), where $p_{m}=p_{m1}=p_{m2}$ and $d_{em}=d_{em1}=d_{em2}$:
  4a $$\begin{aligned} 0\approx&r\left (1-\frac{x_{\text {u}}^{*}}{K}\right )-d_{u}\frac{x_{\text {e}}^{*}}{h_{e}+x_{\text {e}}^{*}}- d_{m1}\left (\frac{d_{ee}}{p_{e}}x_{\text {e}}^{*}+\frac{d_{t}}{p_{e}}x_{\text {u}}^{*}x_{\text {e}}^{*} \right )+d_{m2}c, \end{aligned}$$
  4b $$\begin{aligned} 0=&(a_{1}^{u}+a_{2}^{u})x_{\text {u}}^{*}+p_{m}x_{\text {m}}^{*}\left (1-\frac{x_{\text {m}}^{*}}{M} \right )-d_{em}x_{\text {m}}^{*}. \end{aligned}$$
  Here $x_{\text {m}}^{*}=x_{\text {m1}}^{*}+x_{\text {m2}}^{*}$. Note that (4b) holds true only if the last two terms on the right-hand-side are negative, which is equivalent to $x_{\text {m}}^{*}>(p_{m}-d_{em})M/p_{m}$. Thus, when tumour is present the total macrophages state is higher than the state $x_{\text {m}}^{*}$ obtained for the tumour-free case [see (2b)]. In Fig. 9 we investigate the steady state as we vary (a) the scaling parameter $c\in (0,1)$, (b) the rate $d_{u}$ at which the $\hbox {CD8}^{+}$ T cells eliminate the tumours, and (c) the rate $d_{m1}$ at which the M1 cells eliminate the tumour. Note that an increase in *c* does not change the value of $x_{\text {m}}^{*}$ but it leads to an increase in $x_{\text {u}}^{*}$ (due to a larger M2 cell population). Moreover, while the change in $d_{u}$ affects significantly the long-term tumour size, the change in $d_{m1}$ does not seem to have a significant impact on tumour size when $x_{\text {e}}^{*}$ is small. However, larger $x_{e}^{*}$ values can lead to tumour reduction as we increase $d_{m1}$, and thus we deduce that it is the combined effect of (anti-tumour) macrophages and $\hbox {CD8}^{+}$ T cells that controls tumour growth. As it will be discussed in Appendix A, the complexity of model (1) makes it very difficult to investigate analytically the stability of these steady states, to determine their long-term persistence (although numerical simulations suggest that the state is stable for the parameter values in Table 1; see also Fig. 10b). We would like to emphasise that the existence of this tumour-present virus-absent state is connected to the instability of the previous tumour-free steady state, since this tumour-present state is given by
  5 $$\begin{aligned} x_{\text {u}}^{*}=\frac{K}{r}\left (r -d_{u}\frac{x_{\text {e}}^{*}}{h_{e}+x_{\text {e}}^{*}}-d_{m1}\frac{x^{*}_{\text {m1}}}{h_{m}+x^{*}_{\text {m2}}}+d_{m2}\frac{x^{*}_{\text {m2}}}{h_{m}+x^{*}_{\text {m2}}}\right ), \end{aligned}$$
  which is strictly positive when the inequality (3) is violated. In Fig. 10a we show the regions in the ($r,d_{m2}$) parameter space where the tumour-present virus-absent state exists or not-depending on the sign of equation (5). In Fig. 10b we fix $r=0.374$ and vary $d_{m2}$ within (0.02, 0.2), to be at the border of the stability region for the tumour-free state. We can see that the tumour-free state is stable for $d_{m2}\le 0.075$ and unstable for $d_{m2}>0.075$. When $d_{m2}=0.068$, a second branch of unstable non-zero tumour-present virus-absent states arises; this is shown in Fig. 10(b) on the logarithmic scale. These states stabilise around $d_{m2}=0.159$. We note that for $d_{m2}\in (0.068, 0.091)$ and $d_{m2}\in (0.146, 1.158)$ all eigenvalues of the Jacobian matrix (see Appendix A) are real (corresponding to saddle points), while for $d_{m2}\in [0.092, 0.145]$ two eigenvalues of the Jacobian matrix are complex (corresponding to saddle-focus points). This suggests that another bifurcation occurs for $d_{m2}\approx 0.092$ and $d_{m2}\approx 0.145$, which might lead to oscillations. (We decided not to investigate these possible oscillations here since they occur for $r=0.374\ll 0.924$ the proliferation rate of B16F10 cells.) Moreover, since both states with no virus are unstable for $d_{m2}\in (0.068, 0.159)$ it is likely that the dynamics of the system will approach a steady state where all tumour cells, immune cells and virus particles are present (see below).
(iii) *Tumour-present virus-present immune-present states* To investigate the relation between anti-tumour and anti-viral immune responses, as well as the oncolytic activities of VSV particles, next we focus on the tumour-present virus-present and immune-present (quasi-) steady states ($x_{\text {u}}^{*},x_{\text {i}}^{*},x_{\text {v}}^{*},x_{\text {m1}}^{*},x_{\text {m2}}^{*},x_{\text {e}}^{*}$). (Recall that numerical simulations have shown that the virus is usually eliminated a few days after it is injected, and thus the presence of the virus characterises a quasi steady state.) In Fig. 11a–d we graph, for different parameter values, the surface given by the following equation,
  6 $$\begin{aligned} & \frac{1}{{a_{1}^{v} }}\left( { - x_{{\text{u}}}^{*} (a_{1}^{u} + a_{2}^{u} ) - x_{{\text{m}}}^{*} (p_{m} - d_{{em}} - \frac{{p_{m} }}{M}x_{{\text{m}}}^{*} )} \right) \cdot \left( {d_{u}^{v} \frac{{x_{{\text{e}}}^{*} }}{{h_{e} + x_{{\text{e}}}^{*} }}- d_{{m1}}^{v} \frac{{x_{{\text{e}}}^{*} }}{{p_{e} }}(d_{u} - d_{t} x_{{\text{u}}}^{*} )} \right)- rx_{{\text{u}}}^{*} \left( {1 - \frac{{x_{{\text{u}}}^{*} }}{K}} \right) + d_{u} x_{{\text{u}}}^{*} \frac{{x_{{\text{e}}}^{*} }}{{h_{e} + x_{{\text{e}}}^{*} }} \\ & + d_{{m1}} x_{{\text{u}}}^{*} \frac{{x_{{\text{e}}}^{*} }}{{p_{e} }}(d_{{ee}} - d_{t} x_{{\text{u}}}^{*} ) + d_{{m2}} x_{{\text{u}}}^{*} c = C_{0} ,\;\;{\text{with}}\;C_{0} = \left\{ {\begin{array}{*{20}c} {0,} & {b = 1,} \\ { > 0,} & {b > 1.} \\ \end{array} } \right. \hfill \\ \end{aligned}$$

which connects the cell population states $x_{\text {u}}^{*}$, $x_{\text {e}}^{*}$, and $x_{\text {m}}^{*}=x_{\text {m1}}^{*}+x_{\text {m2}}^{*}$. To investigate how these steady states, and in particular $x_{\text {u}}^{*}$ depends on the density of virus particles (or the density of infected cells), we use a second equation defined in terms of $x_{\text {vi}}^{*}:=x_{\text {v}}^{*}/x_{\text {i}}^{*}$ and $x_{\text {u}}^{*}$, which is graphed in Fig. 11e:

7 $$\begin{aligned} d_{v}x_{\text {vi}}^{*}\frac{x_{\text {u}}^{*}}{h_{u}^{v}+x_{\text {u}}^{*}}-\delta _{i}+\omega -\frac{\delta _{i}b}{x_{\text {vi}}^{*}}=0. \end{aligned}$$

For details on how these equations were obtained, see Appendix A.

Regarding the changes in viral and immune parameters, we notice in Fig. 11 that:

- For the parameter values in Table 1, the tumour-present states exit only for relatively low ratios $x_{\text {vi}}^{*}=x_{\text {v}}^{*}/x_{\text {i}}^{*}<800$; see panel (e). Moreover, the rate $d_{v}$ at which the oncolytic virus infects the tumour cells does not have a significant impact on the persistence of very large tumours (i.e., tumours with $x_{\text {u}}^{*}>10^{8}$); variations in $d_{v}$ are effective for medium-sized tumours and they impact the ratio $x_{\text {vi}}^{*}$.
- Changes in the value of $c=x_{\text {m2}}^{*}/(h_{m}+x_{\text {m2}}^{*})$, which gives an indication on the level of M2 macrophages in the system, does not seem to have a significant effect on $x_{\text {u}}^{*}$; see panel (a). Also note that for the parameter values investigated here, when $x_{\text {e}}^{*}\in (1.2\times 10^{4},1.5\times 10^{4})$ and $x_{\text {m}}^{*}<10^{5}$), there are two possible tumour states $x_{\text {u}}^{*}$: one below $x_{\text {u}}^{*}\approx 10^{7}$, and one above this threshold. This result is particularly interesting since Friberg and Mattson (Friberg and Mattson 1997) suggested that the tumours are detected when their size is above $~10^{7}$ cells. Thus, for relatively similar immune responses ($x_{\text {m}}^{*}$ and $x_{\text {e}}^{*}$) and fixed parameter values, one could obtained large tumours (i.e., $x_{\text {u}}^{*}>10^{9}$ cells) or tumours below the detection threshold. Finally, by comparing the steady-state surfaces generated in sub-panel (a)(ii) for $b=1$ and $b>1$, we conclude that a higher viral burst size ($b>1$) is associated with a larger $\hbox {CD8}^{+}$ T cell population, as well as a smaller tumour size and a smaller macrophage population $x_{\text {m}}^{*}$.
- As expected from the sensitivity analysis, the rate $d_{m1}^{v}$ at which the M1 cells eliminate the virus particles and the virus-infected tumour cells does not have a significant impact on the very large tumour steady states; see panel (b). However, changes in $d_{m1}^{v}$ affect the medium-size tumours, by reducing the range in $x_{\text {e}}^{*}$ over which there is possible to have two tumour sizes (above/below the detection threshold). Also, an increase in $d_{m1}^{v}$ leads to a slight decrease in $x_{\text {e}}^{*}$. This unexpected behaviour is probably the result of the indirect effect of M1 macrophages.
- The rate $d_{u}^{v}$ at which the $\hbox {CD8}^{+}$ T cells eliminate the virus particles and virus-infected tumour cells also has an impact on the small-to-medium size steady states, with lower $d_{u}^{v}$ being associated with lower $x_{\text {e}}^{*}$ states; see panel (c).
- For the parameter values in Table 1, the changes in the rate $d_{u}$ at which the $\hbox {CD8}^{+}$T cells eliminate the uninfected tumour cells do not seem to have a significant effect on small and very large tumours. The only observable effect is on the existence of two different tumour sizes (below and above the detectable threshold of $10^{7}$), which occur also for smaller $x_{\text {e}}^{*}$.

Since it is impossible to obtain closed-form solutions for the tumour-present steady states (due to the high dimensionality of the system (1), and the presence of saturated terms), it is very difficult to investigate analytically the stability of these steady states in terms of various model parameters (see also Appendix A). This is one of the issues associated with complex mathematical models that aim to investigate complex interactions between multiple components of the biological systems.

## Summary and Discussion

In this study we introduced a mathematical model for the investigation of the interactions between melanoma tumour cells, an oncolytic Vesicular Stomatitis Virus (VSV) that was administered twice in 3 days, and innate and adaptive immune responses. We first parametrised the model without the oncolytic virus by fitting it to baseline experimental data in Chen et al. (2011), which focused on the anti-tumour/pro-tumour immune response of the M1 and M2 macrophages. Then, we fixed the tumour and immune-related parameters, and fit the full model with the oncolytic virus (VSV) to the baseline experimental data in Fernandez et al. (2002). To ensure a better model-to-data fit, we incorporated the assumption of higher anti-tumour immune responses following the first viral infection, which leads to the release of TAAs, PAMPs and DAMPs by destroyed tumour cells. For the parameters shown in Table 1, the anti-tumour immune responses following the VSV injection ($d_{u}^{t>15}=0.85$, $d_{m1}^{t>11}=0.29$) are much greater than the anti-tumour immune responses before VSV injection ($d_{u}^{t<15}=0.44$, $d_{m1}^{t<11}=0.01$). However, since the value of the anti-tumour immune response following VSV injection ($d_{u}^{t>15}$, $d_{m1}^{t>11}$) depends on the multitude of fixed tumour and immune parameters (identified through fitting the data in Chen et al. (2011)), and since it is likely that one can find multiple sets of tumour and immune parameters that can fit the data in Chen et al. (2011), we expect that it is possible to find also lower immune responses that fit the data in Fernandez et al. (2002). However, this sort of parameter investigation was not the aim of this current study. Rather, our goal was to investigate whether we can use only one class of mathematical models to reproduce and explain the dynamics suggested by data generated by two different experimental systems, and to further investigate the overall model dynamics. A global parameter optimisation, which can be used to fit at the same time multiple different experiments, will be the subject of a different study. We should emphasise here that despite the possibility of having different sets of parameter values that fit the same data, we would not expect significant changes in the overall dynamics of the system (1) - see also Figs. 5, 6, 7.

Using the model derived in this study we then investigated the different tumour-immune-virus dynamics exhibited by model (1). Thus, through simulations we identified two types of anti-tumour responses, a viral-dominated response and an immune-dominated response (see Fig. 6), which can coexist in the same system and are dependent on different model parameters. Note that the increased viral oncolysis, which leads to tumour elimination in Fig. 6, seems to be immediately followed by an increased anti-tumour immune response that ensures persistent tumour elimination. Despite the numerically identified virus-induced tumour elimination observed in Fig. 6a(i) for large $d_{v}$ values, sensitivity analysis (Fig. 3) showed that variations in $d_{v}$ within $\pm 80\%$ of the identified baseline value could not trigger tumour elimination. It is possible that the baseline value of the virus-infection rate $d_{v}=0.011$ is too low for sufficient virus replication inside tumours. Therefore, unless the tumour-immune-virus microenvironment is perturbed in such a way that allows for higher virus spread, the tumour might never be eliminated. Examples of experimental approaches that have been used to increase viral spread include the use of immunosuppresive chemotherapeutic agents such as cyclophosphamide (Filley and Dey 2017), to reduce the rates at which the immune cells eliminate the virus. Mathematically, this means a reduction in $d_{u}^{v}$ and $d_{m1}^{v}$ baseline values (see Table 1). Numerical simulations, not shown here since they are similar to those shown in Fig. 6a, confirm that a reduction by a factor of 4 of these two rates (i.e., to $d_{u}^{v}=1.1$ and $d_{m1}^{v}=0.375$) leads to tumour elimination due to increased viral replication—thus supporting the importance of viral oncolysis. Moreover, as in Fig. 6, this tumour clearance phenomenon is followed almost immediately by an increase in $\hbox {CD8}^{+}$ T cells and M1 cells levels, thus ensuring that tumour elimination persists for long periods of time. In regard to the anti-tumour immunity following the injection of oncolytic viruses, we have seen in Fig. 5b that multiple VSV injections could eliminate a tumour that is otherwise kept under control by the immune system. One should be aware that larger doses of VSV could have neurotoxic effects (Bridle et al. 2009). Therefore, while multiple VSV injections could lead to better anti-tumour outcomes, one needs to investigate also the optimal level of VSV that is therapeutically safe for the patients.

In the context of question (II) from the Introduction, we showed in Fig. 11 that the macrophages’ plasticity (as quantified by *c*) did not seem to have a big impact on small and large tumours, but might have some impact on medium-size tumours, i.e., tumours around the detection threshold ($x_{\text {u}}^{*}\approx 10^{7}$). In the context of question (III) we noted that the complex interactions between innate and adaptive immunotherapies might lead to intriguing results, where an increase in the anti-viral innate response (described by $d_{m1}^{v}$) has led to a decrease in the number of $\hbox {CD8}^{+}\hbox {T}$ cells that was further associated with a slight increase in the possible tumour sizes. Finally, anti-tumour oncolytic virotherapy (as controlled by $d_{v}$) seemed to have an effect only on medium-to-large tumours.

*Simplicity vs. complexity in mathematical immunology* The complexity of the immune response, and in particular the tumour–immune interactions in the context of macrophages plasticity with multiple cell phenotypes, could lead us to think that incorporating more details into the mathematical models might shed light on the specific aspects of the immune responses that could control tumour growth. For example, one could think that understanding the detailed interactions between the adaptive and innate immunity, or the different aspects of innate immunity, or the saturated vs. linear interactions between tumour cells, immune cells and/or virus particles, might help us find the mechanisms that could control tumour growth. While these specific details could indeed help us investigate (mostly numerically) their role on the overall dynamics of the model, the complexity of the new models that contain large numbers of equations/terms makes them very difficult to be investigated analytically (see Appendix), to draw general conclusions about the importance of specific sets of parameters on tumour control. Moreover, the new models have very large parameter spaces (even after non-dimensionalisation—not shown for this study). Sensitivity analysis could be used to identify the parameters that are most likely to influence model dynamics, but the results are dependent on the baseline parameter values/ranges (obtained through model parametrisation or just guessed).

As shown in Fig. 1, we could have included in system (1) also a Th1–Th2 adaptive immune response (which mirrors the M1-M2 macrophages response), but the model would have been even more complex, and even less informative. Or we could have incorporated other immune cells, such as the NK cells that support oncolytic virotherapies (Diaz et al. 2007a; Bhat and Rommelaere 2015).

In the context of visualising the behaviour of our tumour-immune-virus system, we need to emphasise that the large number of equations in model (1), which underlines the complexity of this model, can lead to some difficulties regarding the calculation of the steady states and their visualisation. In Figs. 8, 9 we graph in 3D two of the simpler steady states exhibited by model (1), as we vary some model parameters. While the 3D-plots are not always very helpful, they might be necessary for a basic understanding of the behaviour of complex models—as shown in Sect. 3.4.

We conclude this discussion by emphasising that such complex mathematical models can only be used to qualitatively investigate the possible dynamics of the system. Moreover, we emphasise that one should strive for simplicity in modelling, as long as the model incorporates all the predominant elements/variables required to answer specific biological questions, and as long as the model can be fitted to available data.

*Model parametrisation* In the published literature, there is very little immunological data that can be used to parametrise mathematical models. The majority of published experimental papers show time series of tumour growth, with extremely few papers showing time series of various immune responses. Moreover, when both such time series can be found, those for the tumour growth are not always obtained under the same conditions (i.e., the same mouse line, the same treatment, etc.), as the time series for the immune responses. To parametrise (relatively complex) mathematical models for tumour–immune interactions, one option is to use multiple data sets obtained in different experimental settings and to further investigate model sensitivity to the identified parameters. In this study we used two different data sets from Fernandez et al. (2002), Chen et al. (2011) where, for consistency, we focused exclusively on the baseline control tumour data (i.e., B16F10 melanoma data obtained in the absence of any external treatment) and macrophages immune data in C57BL/6 mice. However, we should have also parametrised the model using VSV decay data and $\hbox {CD8}^{+}$ T cells data—but we could not find such data. Note that this type of model parametrisation using multiple data sets is somehow similar to the use of parameters taken from various published studies (a very common practice in mathematical immunology, but which leads to more variation in parameter values).

Therefore, for a mathematical model to provide quantitative results that could be used to make predictions which could further inform experiments, it is necessary to parametrise it using sufficient data. Thus more close collaborations between mathematical modellers and experimentalists are necessary to move this field forward.

## Appendix A: Linear Stability of Steady States

The linear stability of the steady states $(x_{\text {u}},x_{\text {i}},x_{\text {v}},x_{\text {m1}},x_{\text {m2}},x_{\text {e}})$ is given by the eigenvalues of the Jacobian matrix (*J*) associated with system (1):

8 $$\begin{aligned} J=\left( \begin{array}{cccccc} a_{11} &{} a_{12} &{} a_{13} &{} a_{14} &{} a_{15} &{} a_{16}\\ a_{21} &{} a_{22} &{} a_{23} &{} a_{24} &{} a_{25} &{} a_{26}\\ 0 &{} a_{32} &{} a_{33} &{} a_{34} &{} a_{35} &{} a_{36}\\ a_{41} &{} a_{42} &{} a_{43} &{} a_{44} &{} a_{45} &{} 0\\ a_{51} &{} 0 &{} a_{53} &{} a_{54} &{} a_{55} &{} 0\\ a_{61} &{} 0 &{} 0 &{} a_{14} &{} a_{15} &{} a_{16}\\ \end{array} \right) , \;\; \text {with}\;\; a_{ij}\ge 0, \end{aligned}$$

and $a_{i,j}$ the terms obtained after differentiating the right-hand-sides of Eq. (1) with respect to the model variables.

At the steady state $(0,0,0,x^{*}_{\text {m1}},x^{*}_{\text {m2}},x^{*}_{\text {e}})$, we obtain $a_{12}=a_{13}=a_{14}=a_{15}=a_{16}=0$, $a_{21}=a_{23}=a_{24}=a_{25}=a_{26}=0$, $a_{31}=a_{34}=a_{35}=a_{36}=0$. By writing down the non-zero terms in this Jacobian matrix one can easily observe that $\det (J-\lambda \mathbb {I}_{6})=0$ (with $\mathbb {I}_{6}$ the $6\times 6$ identity matrix) has the following eigenvalues:

$$\begin{aligned} \lambda _{1}= & {} r-d_{u}\frac{x^{*}_{\text {e}}}{h_{e}+x^{*}_{\text {e}}}-d_{m1}\frac{x^{*}_{\text {m1}}}{h_{m}+x^{*}_{\text {m2}}}+d_{m2}\frac{x^{*}_{\text {m2}}}{h_{m}+x^{*}_{\text {m2}}},\\ \lambda _{2}= & {} -\delta _{i}-d_{u}^{v}\frac{x^{*}_{\text {e}}}{h_{e}+x^{*}_{\text {e}}}-d_{m}^{v}\frac{x^{*}_{\text {m1}}}{h_{m}+x^{*}_{\text {m2}}}<0,\\ \lambda _{3}= & {} -\omega -d_{u}^{v}\frac{x^{*}_{\text {e}}}{h_{e}+x^{*}_{\text {e}}}-d_{m1}^{v}\frac{x^{*}_{\text {m1}}}{h_{m}+x^{*}_{\text {m2}}}<0,\\ \lambda _{6}= & {} -d_{ee}<0. \end{aligned}$$

From here is it easily observed that asymptotic stability would require $\lambda _{1}<0$ (see Equation (3)). The other two eigenvalues ($\lambda _{4,5}$) are given by the roots of a quadratic:

$$\begin{aligned} \lambda _{4,5}=\frac{1}{2}\left ( A\pm \sqrt{A^{2}-4B} \right ), \end{aligned}$$

where

$$\begin{aligned} A=-p_{m}+d_{em}-(r_{m1}^{0}+r_{m2}^{0})<0 \;\;\text {since}\;\; p_{m}>d_{em} \;\; (\text {for} \; x^{*}_{\text {m}}\; \text {to exist}), \end{aligned}$$

and

$$\begin{aligned} B= & {} \Big [ p_{m}\Big (1-\frac{x^{*}_{\text {m}}}{M}\Big )-d_{em} \Big ]^{2} - \Big [p_{m}\Big (1-\frac{x^{*}_{\text {m}}}{M}\Big )-d_{em} \Big ]\Big [p_{m}\frac{x^{*}_{\text {m}}}{M}+r_{m1}^{0}+r_{m2}^{0} \Big ] \\&+\frac{p_{m}x^{*}_{\text {m}}}{M}(r_{m1}^{0}+r_{m2}^{0}). \end{aligned}$$

We note that $B>0$ since $p_{m}(1-\frac{x_{\text {m}}^{*}}{M})-d_{em}=0$ (from the definition of $x_{\text {m}}^{*}$; see Eq. (2b)). Since $A<0$ and $B>0$ we deduce that $\lambda _{4,5}<0$. Therefore, the stability of the tumour-free state is given exclusively by the sign of $\lambda _{1}$, as requested by Equation (3).

### Remark 3

Note that to calculate the tumour-free virus-free steady state $(0,0,0,x_{\text {m1}}^{*},x_{\text {m2}}^{*},x_{\text {e}}^{*})$ given by Equations (2), we used the steady-state Equation (1f) to obtain $x_{\text {e}}^{*}$, and the steady-state Equations (1d) and (1e) (in which we substituted the expression for $x_{\text {m}}^{*}=x_{\text {m1}}^{*}+x_{\text {m2}}^{*}$, with $x_{\text {m}}^{*}$ satisfying (2b)) to obtain $x_{\text {m1}}^{*}$ and $x_{\text {m2}}^{*}$. For example,

$$\begin{aligned} (1d)\Rightarrow\, & {} 0=x_{\text {m1}}^{*}\Big (p_{m}\big (1-\frac{x_{\text {m}}^{*}}{M} \big )-d_{em} \Big )-x_{\text {m1}}^{*}r_{m1}^{0}+(x_{\text {m}}^{*}-x_{\text {m1}}^{*})r_{m2}^{0}\\\Leftrightarrow\, & {} 0=x_{\text {m1}}^{*}\underbrace{\Big (0\Big )}_{Eq.(2b)}-x_{\text {m1}}^{*}r_{m1}^{0}+(x_{\text {m}}^{*}-x_{\text {m1}}^{*})r_{m2}^{0} \\\Rightarrow\, & {} x_{\text {m1}}^{*}=\frac{x_{\text {m}}^{*} r_{m2}^{0}}{r_{m1}^{0}+r_{m2}^{0}}. \end{aligned}$$

In a similar manner one can use the steady state Equation (1e) to obtain the expression for $x_{\text {m2}}^{*}$.

At the steady state $(x_{\text {u}}^{*},0,0,x^{*}_{\text {m1}},x^{*}_{\text {m2}},x^{*}_{\text {e}})$, given implicitly by Eq. (4), we obtain $a_{12}=0$, $a_{21}=a_{24}=a_{25}=a_{26}=0$, $a_{31}=a_{34}=a_{35}=a_{36}=0$. The eigenvalues $\lambda$ of the Jacobian matrix satisfy a 6th-order polynomial, which cannot be solved explicitly (to identify the combinations of parameters that control the stability of the steady states). Moreover, as we can see in Fig. 9, for fixed parameter values there seem to be an infinite number of possible steady states.

The coefficient terms in this 6th-order polynomial are even more complicated if we consider the tumour-present virus-present immune-present states $(x_{\text {u}},x_{\text {i}},x_{\text {v}},x_{\text {m1}},x_{\text {m2}},x_{\text {e}})$. Therefore, the complexity of the model (and in particular the presence of nonlinear saturated terms) renders almost impossible the classical linear stability analysis (although stability can be calculated numerically for specific parameter values, as shown in Fig. 10).

### Remark 4

Not only that we cannot investigate the stability of the tumour-present virus-present immune-present steady stats, but even finding closed-form equations for these states is challenging. To obtain the implicit Eqs. (6)–(7) that describe these states, we started with Equation (1f) from which we obtained

$$\begin{aligned} \frac{x_{\text {m1}}^{*}}{h_{m}+x_{\text {m2}}^{*}}=\frac{x_{\text {e}}^{*}}{p_{e}}\big (d_{ee}-d_{t}x_{\text {u}}^{*} \big ) \end{aligned}$$

Then, adding (1b)+(1c) in which we substitute the above expression leads to

9 $$\begin{aligned} d_{v}x_{\text {v}}^{*}\frac{x_{\text {u}}^{*}}{h_{u}^{v}+x_{\text {u}}^{*}}=-\delta _{i}x_{\text {i}}^{*}(b-1)-(x_{\text {i}}^{*}+x_{\text {v}}^{*})\Big (d_{u}^{v}\frac{x_{\text {e}}^{*}}{h_{e}+x_{\text {e}}^{*}}-d_{m1}^{v}\frac{x_{\text {e}}^{*}}{p_{e}}(d_{ee}-d_{t}x_{\text {u}}^{*}) \Big ). \end{aligned}$$

For $b=1$ the first term on the right-hand-side vanishes. If $b>1$, we can denote this first term by $C_{0}$.

If we now focus on the steady-state Eq. (1a), and solve for the same term $d_{v}x_{\text {v}}^{*}x_{\text {u}}^{*}/(h_{u}^{v}+x_{\text {u}}^{*})$ while denoting by $c=x_{\text {m2}}^{*}/(h_{m}+x_{\text {m2}}^{*})\in (0,1)$, we obtain :

10 $$\begin{aligned} d_{v}x_{\text {v}}^{*}\frac{x_{\text {u}}^{*}}{h_{u}^{v}+x_{\text {u}}^{*}}=rx_{\text {u}}^{*}\Big (1-\frac{x_{\text {u}}^{*}}{K} \Big )-d_{u}x_{\text {u}}^{*}\frac{x_{\text {e}}^{*}}{h_{e}+x_{\text {e}}^{*}}-d_{m1}x_{\text {u}}^{*}\frac{x_{\text {e}}^{*}}{p_{e}}(d_{ee}-d_{t}x_{\text {u}}^{*})+d_{m2}x_{\text {u}}^{*}c. \end{aligned}$$

Equating (9) and (10) leads to the following equation (which combines (1a), (1b), (1c) and (1f)):

11 $$\begin{aligned}&(x_{\text {i}}^{*}+x_{\text {v}}^{*})\Big [d_{u}^{v}\frac{x_{\text {e}}^{*}}{h_{e}+x_{\text {e}}^{*}}-d_{m1}^{v}\frac{x_{\text {e}}^{*}}{p_{e}} (d_{ee}-d_{t}x_{\text {u}}^{*}) \Big ]=rx_{\text {u}}^{*}\Big (1-\frac{x_{\text {u}}^{*}}{K} \Big )-d_{u}x_{\text {u}}^{*}\frac{x_{\text {e}}^{*}}{h_{e}+x_{\text {e}}^{*}} \\&\quad -d_{m1}x_{\text {u}}^{*}\frac{x_{\text {e}}^{*}}{p_{e}}(d_{ee}-d_{t}x_{\text {u}}^{*})+d_{m2}x_{\text {u}}^{*}c+C_{0}. \end{aligned}$$

On the other hand, combining the steady-state Eqs. (1d) and (1e) leads to

12 $$\begin{aligned} (x_{\text {i}}^{*}+x_{\text {v}}^{*})=\frac{1}{a_{1}^{v}}\Big [-x_{\text {u}}^{*}(a_{1}^{u}+a_{2}^{u})-x_{\text {m}}^{*}\big (p_{m}-d_{em}-\frac{p_{m}}{M}x_{\text {m}}^{*} \big ) \Big ]. \end{aligned}$$

Substituting (12) into (11) leads to Equation (6).

To obtain (7), we eliminate the similar terms in (1b) and (1c) that contain $x_{\text {e}}^{*}$ and $x_{\text {m1}}^{*}$, namely $d_{u}^{v}x_{\text {e}}^{*}/(h_{e}+x_{\text {e}}^{*})+d_{m1}^{v}x_{\text {m1}}^{*}/(h_{m}+x_{\text {m2}}^{*})$. This leads to

13 $$\begin{aligned} d_{v}\frac{x_{\text {v}}^{*}}{x_{\text {i}}^{*}}\frac{x_{\text {u}}^{*}}{h_{u}^{v}+x_{\text {u}}^{*}}-\delta _{i}=\delta _{i}b \frac{x_{\text {i}}^{*}}{x_{\text {v}}^{*}}-\omega . \end{aligned}$$

Denoting by $x_{\text {vi}}^{*}:=x_{\text {v}}^{*}/x_{\text {i}}^{*}$, the above equation reduces to (7).
